# Prognostic value of preoperative nutritional status for postoperative moderate to severe acute kidney injury among older patients undergoing coronary artery bypass graft surgery: a retrospective study based on the MIMIC-IV database

**DOI:** 10.1080/0886022X.2024.2429683

**Published:** 2024-12-01

**Authors:** Peng Bao, Peng Qiu, Tao Li, Xue Lv, Junyu Wu, Shaojie Wu, Hao Li, Zhiping Guo

**Affiliations:** a Department of Cardiac Rehabilitation, Fuwai Central China Cardiovascular Hospital, Fuwai Central China Hospital of Zhengzhou University, Zhengzhou, Henan, China; b Department of Rehabilitation, the First Affiliated Hospital of Wenzhou Medical University, Wenzhou, Zhejiang, China; c Health Management Center, Henan Key Laboratory of Chronic Disease Management, Fuwai Central China Cardiovascular Hospital, Fuwai Central China Hospital of Zhengzhou University, Zhengzhou, Henan, China; d Ministry of Cadres Health, Henan Provincial People’s Hospital, Zhengzhou, Henan, China; e School of Physical Education, Shanghai University of Sport, Shanghai, China; f Henan Key Laboratory of Chronic Disease Management, Fuwai Central China Cardiovascular Hospital, Fuwai Central China Hospital of Zhengzhou University, Henan Cardiovascular Disease Center, Zhengzhou, Henan, China

**Keywords:** Acute kidney injury, CABG, malnutrition, prediction model

## Abstract

**Objective:**

To investigate the association between preoperative nutritional scores and moderate-to-severe acute kidney injury (AKI) after coronary artery bypass graft (CABG) surgery and the predictive significance of nutritional indices for moderate to severe AKI.

**Methods:**

This study retrospectively included older patients underwent CABG surgery from the Medical Information Mart for Intensive Care (MIMIC) database. Nutritional scores were calculated by the Geriatric Nutritional Risk Index (GNRI) and the Prognostic Nutritional Index (PNI), respectively. Moderate-to-severe injury was determined by KDIGO criteria. Logistic regression, subgroup analysis, and restricted cubic splines were utilized to investigate the association. The predictive value was also assessed by the area under the curve (AUC), net reclassification index (NRI), and integrated discrimination improvement (IDI).

**Results:**

A total of 1,007 patients were retrospectively included, of which 100 (9.9%) and 380 (37.7%) had malnutrition calculated by GNRI and PNI scores. The incidence of moderate-to-severe AKI was 524 (52.0%). After adjustment for selected risk factors, worse nutritional scores were associated with a higher incidence of moderate-to-severe AKI (*P*_GNRI_<0.001; *P*_PNI_=0.001). Integrating these indices into different base models improves their performance, as manifested by significant improvements in AUCs and NRIs (*p* < 0.05).

**Conclusion:**

Worse preoperative nutritional status was associated with an elevated risk of postoperative moderate-to-severe AKI. Integrating these indices into base models improve their predictive performance. These results highlight the importance of assessing nutritional status among older patients had CABG surgery.

## 1. Introduction

Acute kidney injury (AKI) after coronary artery bypass graft (CABG) surgery is a widespread complication with an incidence between 19% and 28% [1–3], and it is particularly common in older patients [4]. In different types of cardiac surgery, the AKI incidence in patients with CABG surgery was lower compared to those who had aortic surgery or valve surgery [5]. Its severe form is independently associated with elevated morbidity and mortality in the cardiac surgery patients [6,7]. The mortality risk associated with AKI following cardiac surgery persists for 10 years and is independent of additional risk factors, even for patients who have recovered their kidneys completely [8]. Moderate-to-severe (stage 2 to 3) AKI has attached greater attention due to the complexity of its treatment. Timely recognition of AKI is important in order to initiate a comprehensive treatment strategy to minimize further damage and decreased associated mortality [9]. However, the pathogenesis of AKI following cardiac surgery is complicated and incompletely understood [10], early identifying predictive factors associated with AKI is urgent.

Malnutrition involves multi-dimensional conditions characterized by a reduction of the body fat and/or muscle mass as a result of insufficient nutrient intake or utilization [11]. The incidence of malnutrition in the elderly is 25% [12]. Worse nutritional status is associated with many unfavorable outcomes in older patients, including delirium, in-hospital mortality, re-hospitalizations rate and long-term mortality [13–15]. The assessment methods based on the questionnaires to screen nutritional status have previously been utilized extensively. However, these approaches are inappropriate for geriatrics because of the recall bias and communication problems [13,14]. To overcome these limitations of questionnaires, some objective nutritional indicators based on routine laboratory tests have been validated and utilized, including the geriatric nutritional risk index (GNRI) and prognostic nutritional index (PNI). The sensitivity and specificity of the GNRI for assessing the malnutrition were 62.2% and 97.7% [16]. As for the PNI, the sensitivity and specificity for evaluating the malnutrition were 74.1%–80.8% and 66.4%–88.2% due to different subjects [17,18]. In addition, preoperative poor nutritional status calculated by GNRI or PNI was significantly associated with elevated AKI incidence [19–21], and higher mortality in cardiac surgery patients [22–24]. However, these studies failed to sufficiently take into account the complexity of nutritional scores being both a categorical and a continuous variable, nor did they consider the potential predictive value of GNRI and PNI. Small sample sizes are issues that cannot be disregarded. In addition, older patients rarely got much attention in patients only underwent CABG surgery.

The purpose of this study is to completely investigate the association between the preoperative nutritional status, as evaluated by GNRI and PNI, and moderate-to-severe AKI following CABG surgery among older patients. Furthermore, we also determine whether GNRI and PNI can improve the model performance based on the traditional risk factors.

## 2. Methods

### 2.1. Study design

The retrospective study used data derived from the Medical Information Mart for Intensive Care IV (MIMIC-IV, version 2.2) database, encompassing detailed medical information from patients admitted to the Intensive Care Units (ICUs) of Beth Israel Deaconess Medical Center from 2008 to 2019. The author passed the Collaborative Institutional Training Initiative (CITI) examination, received a certification (ID number: 60692864), and then accessed this database, which obviated the need for individual patient consent.

**Figure 1.**
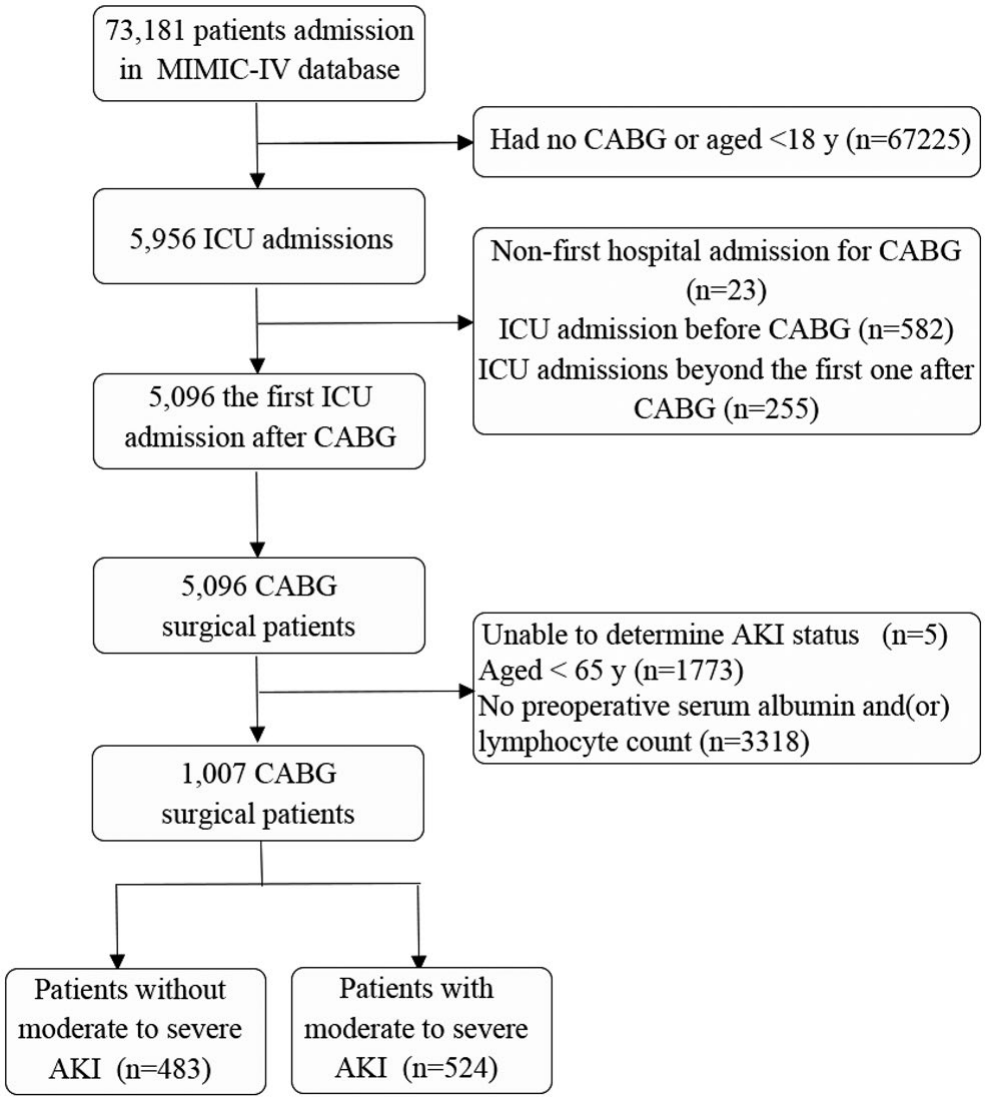
Flow diagram for patient selection. ICU, intensive care unit; MIMIC, medical information mart for intensive care; CABG, coronary artery bypass grafting; AKI, acute kidney injury.

This study included 5,956 patients who were hospitalized to the Cardiac Surgery ICU following CABG in the MIMIC IV database. The following exclusion criteria were applied for patients: (1) Not first hospital admission for CABG; (2) ICU admission before CABG; (3) ICU admission beyond the first one after CABG; (4) Age < 65 years; (5) Failure to evaluate postoperative AKI stage; (6) Lossing key information of preoperative serum albumin and lymphocyte counts. After applying these criteria, the final sample comprised 1,007 patients (Figure 1).

### 2.2. Data collection

The study involved the extraction of comprehensive patient data, including demographics (gender, age, heigh, weight, and body mass index [BMI]) and comorbidities (congestive heart failure, peripheral vascular disease, diabetes, hypertension, chronic pulmonary disease, chronic liver disease, stroke, chronic kidney disease, cerebrovascular disease, and Charlson comorbidity index). The definition of chronic kidney disease is an abnormality in the structure or function of the kidney that persists for more than three months and has an impact on health [25]. Additionally, preoperative clinical status indicators such as the Sequential Organ Failure Assessment (SOFA) score, Glasgow Coma Scale (GCS), heart rate, oxyhemoglobin saturation (SPO_2_), mean blood pressure (MBP), use of intra-aortic balloon pump (IABP), ventilators, and vasopressors were carefully documented. Preoperative laboratory parameters, including albumin, serum creatinine (Scr), hematocrit, blood urea nitrogen (BUN), potassium, sodium, bicarbonate, and lymphocyte counts, were also recorded. Lastly, the first postoperative laboratory values for creatinine, BUN, sodium, potassium, and bicarbonate, along with the duration between CABG surgery completion and the initial renal function measurement, were systematically collected.

### 2.3. Outcome assessments

The primary outcome was the severity of AKI within seven days following CABG operation, classified into stages 0, 1, 2, or 3 according to the Kidney Disease Improving Global Outcomes (KDIGO) criteria [26]. The highest Scr level recorded within this period determined the AKI stage, with stage 2 to 3 considered moderate-to-severe AKI. The baseline Scr level was evaluated using the most recent measurements prior to surgery. Secondary outcomes encompassed the length of stay (LOS) in ICU and in hospital, as well as hospital mortality, 30-day and 90-day mortality.

### 2.4. Nutritional evaluation and categorization

The GNRI was computed utilizing the formula: 1.489 × serum albumin (g/L) + 41.7 × (actual body weight [kg]/ideal body weight [kg]). In addition, this formula for calculating the ideal weight varies according to gender: 0.75 × height (cm) − 62.5 for males and 0.60 × height (cm) − 40 for females. Based on GNRI scores, patients were categorized into two groups reflecting their risk of malnutrition: no nutritional risk group (GNRI > 98) and nutritional risk group (GNRI ≤ 98) [27].

The PNI was evaluated by the formula: serum albumin (g/L) + 5 × total lymphocyte count (×10^9^/L), dividing patients into following two groups: no nutritional risk group (PNI ≥ 48) and nutritional risk group (PNI <48) [19,24,28].

### 2.5. Statistical analysis

The R software (version 4.2.1) and SPSS (version 25.0) were utilized to carry out the significant difference analysis. The Shapiro–Wilk test was utilized across all continuous variables to determine whether it is normally or abnormally distributed. They were expressed as mean ± standard deviation or medians (interquartile ranges [IQR]) and compared by the Mann–Whitney U-test. Afterwards, categorical data were stated as frequencies (percentages) and assessed utilizing the chi-square test or Fisher exact test. The relationship between the GNRI and the PNI was depicted by a Venn diagram. The Spearman’s correlation coefficients (r) were utilized to investigate the correlation between the GNRI and PNI.

The association between nutritional condition and the incidence of moderate to severe AKI after CABG surgery was explored through logistic regression, treating nutritional indices as both continuous and categorical data. Covariables that were associated with moderate-to-severe AKI following CABG surgery in the adjusted model were calculated by multivariate logistic regression. To eliminate multicollinearity, components used in calculating malnutrition scores, such as weight, height, BMI, lymphocyte counts and serum albumin were omitted from the multivariate regression analysis. An initial univariate analysis was followed by a multivariate approach to adjust for screened confounders. Three multivariate models were developed to validate the association: Model 1 adjusted by demographic factors including gender, congestive heart failure, and chronic pulmonary disease; Model 2 expanded on Model 1 by incorporating preoperative conditions such as the SOFA score, MBP, preoperative hematocrit, and BUN; Model 3 further included postoperative creatinine and BUN concentrations. These results were reported as odds ratios (ORs) and 95% confidence intervals (CIs).

The multivariable association between preoperative nutritional assessments and the incidence of moderate to severe AKI following CABG surgery was also elucidated using restricted cubic splines (RCS), which necessitated the treatment of the GNRI and PNI exclusively as continuous variables for statistical analysis. The RCS could not be displayed when the nutritional indices were regarded as categorical variables. Similarly, this approach was utilized to determine the optimal cutoff points of GNRI and PNI [29]. In order to further explain the importance of every categorical variable to the moderate-to-severe AKI after CABG surgery, subgroup analyses were conducted. There was an interaction effect when the *p* for interaction across subgroups < 0.05 [30].

Furthermore, the incremental predictive utility of these nutritional indices for moderate-to-severe AKI after CABG surgery was assessed by integrating them into three base models (Model 1, 2, and 3), thereby generating refined predictive models. The discriminative capability of these nutritional metrics was determined through the area under the curve (AUC), the net reclassification index (NRI), and integrated discrimination improvement (IDI). In addition, calibration plots, utilizing bootstrapping methods with 1000 resamples, were utilized to compare the predicted risks with the observed frequency, and Brier scores < 0.25 demonstrated goodness of fits. Decision curve analyses were subsequently applied to ascertain the clinical utility.

The minimum sample size was determined by the package ʻpmsamplesizeʼ in the R software [31]. The minimum sample size was 915 individuals, according to the AUC of 0.79, 28.7% incidence of postoperative moderate to severe AKI and 14 variables [21,32]. The *p*-value < 0.05 denotes statistical significance.

## 3. Results

### 3.1. Clinical characteristics

The selection process for patients was depicted, with a total of 1007 patients retrospectively included (Figure 1). As the result, the median age was 73.46 years (IQR: 69.1–79.02), with a predominance of male patients (73.1%). The median BMI was recorded at 28.96 (IQR: 25.89–32.49). Hypertension, diabetes, and congestive heart failure emerged as the most prevalent comorbidities. The median lengths of stay (LOS) in ICU and hospital were 1.98 days (IQR: 1.26–3.22) and 8.05 days (IQR: 5.74–11.45), respectively. Observed mortality rates for the hospital, 30-day, and 90-day periods were 1.3%, 1.6%, and 1.8%, respectively.

Among all eligible patients, 798 (79.2%) had AKI following CABG surgery within 7 days, with 27.2% at stage 1 (*n* = 274), 44.7% at stage 2 (*n* = 450), and 7.3% at stage 3 (*n* = 74). The incidence of moderate-to-severe AKI (stages 2–3) was 524 (52.0%). The characteristics of the study cohort were showed, ranging from global to stratified data (Table 1 and Table S1). Patients with moderate-to-severe AKI typically exhibited a higher BMI, a shorter LOS in ICU and in hospital, and a higher prevalence of comorbidities, including congestive heart failure, hypertension, chronic pulmonary disease, and diabetes. These patients also presented a lower MBP and hematocrit levels prior to CABG surgery, whereas the SOFA score, utilization rate of IABP, the creatinine, and BUN were elevated compared to those without moderate to severe AKI. Furthermore, creatinine and BUN levels were higher in the first renal function test after CABG surgery. Additionally, the score of GNRI was higher and the metric of PNI was significantly lower among patients with moderate-to-severe AKI.

**Table 1.** Baseline characteristics of study participants.

	overall	no moderate to severe AKI	moderate to severe AKI	OR (95% CI)	*p* value
	1007	483	524		
Gender (Male)	736 (73.1)	371 (76.8)	365 (69.7)	0.69 (0.52-0.92)	0.013
Age (year)	73.46 [69.1–79.02]	72.80 [68.91, 78.62]	74.01 [69.21, 79.51]	1.02 (1-1.04)	0.128
BMI (kg/m^2^)	28.96 [25.89–32.49]	27.64 [24.84, 30.97]	30.20 [27.20, 33.91]	1.12 (1.09-1.15)	<0.001
Height (cm)	170.00 [165.00, 178.00]	170.00 [165.00, 178.00]	172.41 [164.29, 178.00]	1.01 (1-1.02)	0.157
Weight (kg)	84.05 [74.10–96.45]	80.00 [71.50, 90.97]	88.10 [77.00, 101.30]	1.04 (1.03-1.04)	<0.001
Comorbid disease					
Charlson comorbidity index	5.00 [4.00–7.00]	5.00 [4.00, 6.00]	5.00 [4.00, 7.00]	1.17 (1.1-1.25)	<0.001
Congestive heart failure	287 (28.5)	98 (20.3)	189 (36.1)	2.22 (1.67-2.95)	<0.001
Peripheral vascular disease	153 (15.2)	71 (14.7)	82 (15.6)	1.08 (0.76-1.52)	0.74
Hypertension	588 (58.4)	298 (61.7)	290 (55.3)	0.77 (0.6-0.99)	0.048
Chronic pulmonary disease	212 (21.1)	86 (17.8)	126 (24.0)	1.46 (1.07-1.99)	0.019
Diabetes	427 (42.4)	186 (38.5)	241 (46.0)	1.36 (1.06-1.75)	0.019
Chronic liver disease	16 (1.6)	4 (0.8)	12 (2.3)	2.81 (0.9-8.76)	0.109
Stroke	75 (7.4)	35 (7.2)	40 (7.6)	1.06 (0.66-1.7)	0.909
Chronic kidney disease	227 (22.5)	92 (19.0)	135 (25.8)	1.47 (1.09-1.99)	0.013
Cerebrovascular disease	131 (13.0)	63 (13.0)	68 (13.0)	0.99 (0.69-1.44)	1
GCS	14.00 [13.00–15.00]	14.00 [14.00, 15.00]	14.00 [12.00, 15.00]	0.97 (0.94-1)	0.007
SOFA	6.00 [4.00–8.00]	5.00 [4.00, 7.00]	6.00 [4.00, 9.00]	1.15 (1.1-1.2)	<0.001
Heart rate (beat)	82.00 [77.00, 87.00]	82.00 [77.00, 87.00]	81.00 [77.00, 88.00]	1 (0.99-1.02)	0.968
MBP (mmHg)	73.00 [69.00, 76.00]	74.00 [70.00, 77.00]	72.00 [69.00, 75.00]	0.94 (0.92-0.96)	<0.001
SPO_2_ (%)	98.00 [97.00, 99.00]	98.00 [97.00, 99.00]	98.00 [97.00, 99.00]	1.01 (0.92-1.11)	0.872
IABP	63 (6.3)	20 (4.1)	43 (8.2)	2.07 (1.2-3.57)	0.011
Ventilator	872 (86.6)	414 (85.7)	458 (87.4)	1.16 (0.8-1.66)	0.488
Vasopressor	876 (87.0)	411 (85.1)	465 (88.7)	1.38 (0.95-2)	0.104
Preoperative laboratories					
Albumin (mg/dL)	4.00 [3.60–4.30]	4.00 [3.70–4.40]	3.90 [3.50–4.20]	0.5 (0.39-0.65)	<0.001
Creatinine (mg/dL)	1.00 [0.90–1.30]	1.00 [0.90, 1.20]	1.10 [0.90, 1.30]	1.51 (1.2-1.9)	0.004
Hematocrit (%)	37.60 [33.20–41.10]	38.50 [34.50–41.50]	36.35 [31.80–40.50]	0.96 (0.94-0.98)	<0.001
BUN (mg/dL)	20.00 [16.00–26.00]	20.00 [16.00, 25.00]	21.00 [16.00, 27.00]	1.02 (1.01-1.03)	0.063
Sodium (mmol/L)	139.00 [137.00–141.00]	139.00 [137.00, 141.00]	139.00 [137.00, 141.00]	0.99 (0.95-1.02)	0.437
Potassium (mmol/L)	4.20 [4.00–4.50]	4.20 [4.00, 4.50]	4.20 [4.00, 4.50]	0.97 (0.73-1.29)	0.36
Bicarbonate (mmol/L)	26.00 [24.00–28.00]	26.00 [24.00, 28.00]	26.00 [24.00, 28.00]	0.97 (0.94-1.01)	0.215
Lymphocyte (10^3^/μL)	1.63 [1.24–2.12]	1.68 [1.29, 2.18]	1.57 [1.19, 2.00]	0.83 (0.71-0.96)	0.002
GNRI	114.27 (12.57)	112.8 (11.46)	115.62 (13.37)	1.02 (1.01-1.03)	0.001
GNRI ≤ 98	100 (9.9)	49 (10.1)	51 (9.7)	1.05 (0.69-1.58)	0.91
PNI	48.39 [44.15–52.64]	49.45 [45.64, 53.91]	47.02 [42.83, 51.65]	0.95 (0.93-0.97)	<0.001
PNI < 48	480 (47.7)	194 (40.2)	286 (54.6)	0.56 (0.43-0.72)	<0.001
Postoperative laboratories					
Creatinine (mg/dL)	0.90 [0.70–1.10]	0.90 [0.70, 1.00]	0.90 [0.70, 1.10]	1.83 (1.37-2.45)	<0.001
BUN (mg /dL)	17.00 [14.00–21.00]	16.00 [13.00, 20.00]	18.00 [14.00, 23.00]	1.03 (1.02-1.05)	<0.001
Sodium (mmol/L)	139.00 [137.00–141.00]	139.00 [137.00, 141.00]	139.00 [137.00, 141.00]	0.97 (0.93-1.01)	0.089
Potassium (mmol/L)	4.30 [3.90–4.60]	4.20 [3.90, 4.60]	4.30 [4.00, 4.70]	1.23 (0.98-1.55)	0.069
Bicarbonate (mmol/L)	23.00 [21.00–24.00]	23.00 [21.00–24.00	23.00 [21.00–24.00	0.98 (0.93-1.04)	0.931
Time interval (h)	3.50 [2.48–4.86]	3.39 [2.48, 4.76]	3.57 [2.50, 4.99]	1.02 (0.95-1.09)	0.364
outcome					
LOS in ICU (day)	1.98 [1.26–3.22]	1.35 [1.16, 2.25]	2.37 [1.37, 4.17]	1.34 (1.24-1.45)	<0.001
LOS in hospital (day)	8.05 [5.74–11.45]	7.05 [5.30, 9.91]	9.13 [6.61, 12.70]	1.11 (1.08-1.14)	<0.001
Hospital mortality (%)	13 (1.3%)	3 (0.62%)	10 (1.91%)	3.11 (0.85-11.38)	0.126
Mortality 30‐day (%)	16 (1.6%)	5 (1.04%)	11 (2.10%)	2.05 (0.71-5.94)	0.273
Mortality 90‐day (%)	38 (3.8%)	12 (2.48%)	26 (4.96%)	2.05 (1.02-4.11)	0.058

Values are expressed as median (interquartile range) or mean (standard deviation) and frequencies (percentages). AKI, acute kidney injury; BMI, body mass index; GCS, Glasgow coma scale; SOFA, sequential organ failure assessment; MBP, mean blood pressure; SPO_2_, oxyhemoglobin saturation by pulse oximetry; IABP, intra-aortic balloon pump; GNRI, geriatric nutritional risk index; PNI, prognostic nutritional index; BUN, blood urea nitrogen; LOS, length of stay.

### 3.2. Prevalence and distribution of malnutrition

**Figure 2.**
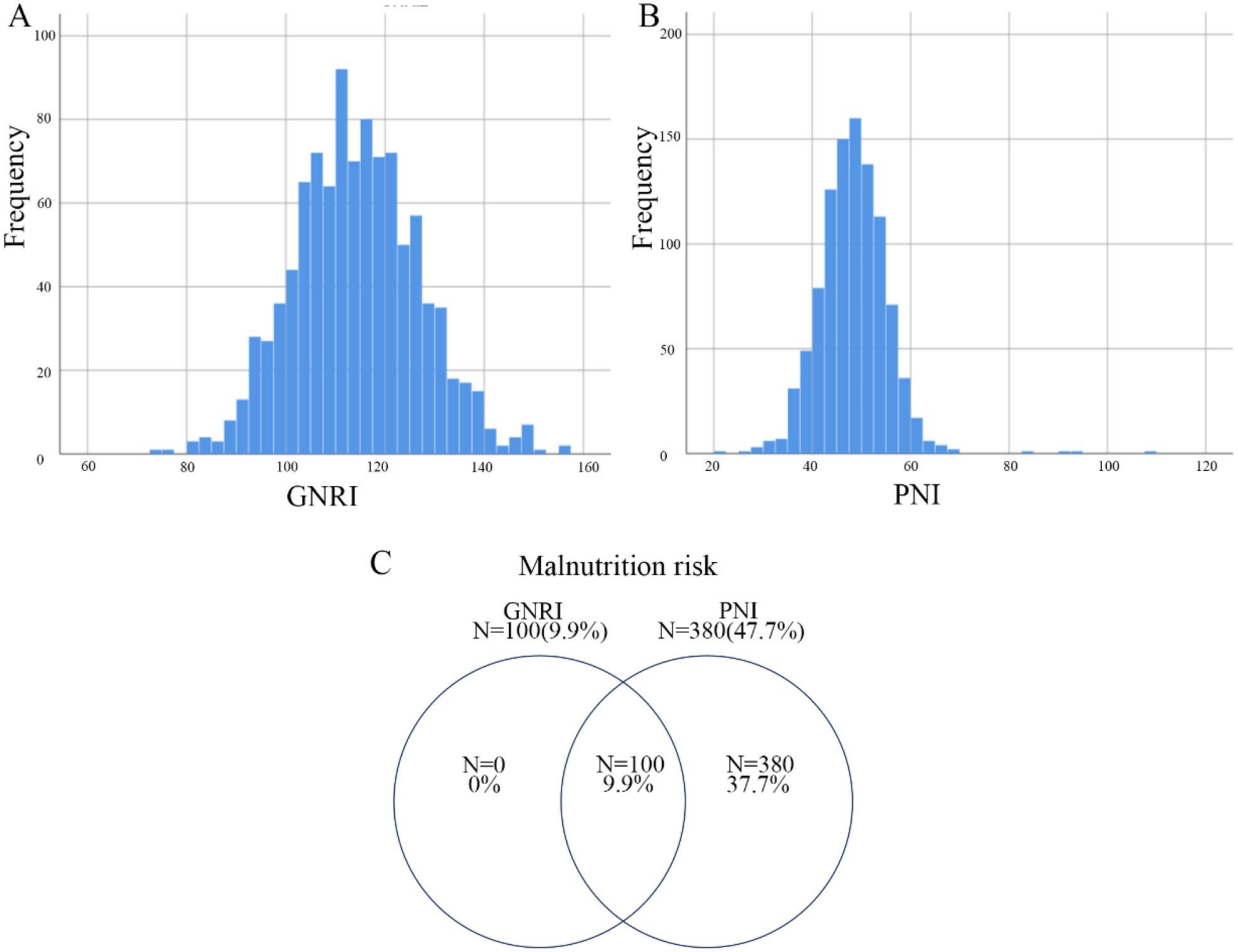
Population distribution and incidence of malnutrition. Histograms show the population distribution of GNRI (a), and PNI (B). Venn diagram of malnutrition risk assessed by the GNRI and PNI(C). The frequency of malnutrition is shown by the frequencies and percentages outside of each circle. The area where two circles overlap indicates the frequency and percentage of a malnutrition diagnosis with GNRI and PNI coincide. GNRI, geriatric nutritional risk index; PNI, prognostic nutritional index.

The distributions of GNRI and PNI were indicated. The prevalence of malnutrition among the patients was found to be 9.9% when assessed by the GNRI and 47.9% when evaluated through the PNI (Figure 2). There was a significant correlation between these two indicators (*p* < 0.001, *r* = 0.541). Patients with malnutrition evaluated by GNRI and PNI were older, had a lower BMI, a worse SOFA score, and a higher utilization rate of IABP compared with patients in normal nutritional status. In addition, they tend to have a higher probability of having congestive heart failure and a lower percentage of combining hypertension (Tables S2–S3).

**Table 2.** Univariable and multivariable analyses of GNRI and PNI to predict moderate to severe AKI after CABG.

	Univariable analysis		Multivariable analysis model 1		Multivariable analysis model 2		Multivariable analysis model 3	
	OR (95%)	*P*	OR (95%)	*P*	OR (95%)	*P*	OR (95%)	*P*
Nutritional indices as continuous variables								
GNRI	1.018 (1.008–1.029)	<0.001	1.022 (1.011–1.033)	<0.001	1.03 (1.018–1.041)	<0.001	1.027 (1.016-1.039)	<0.001
PNI	0.947 (0.928–0.966)	<0.001	0.955 (0.936–0.974)	<0.001	0.964 (0.944–0.984)	0.001	0.963 (0.943-0.984)	0.001
Nutritional indices as category variables								
GNRI > 98	Ref.		Ref.		Ref.		Ref.	
GNRI ≤ 98	0.955 (0.632–1.444)	0.827	0.803 (0.524–1.23)	0.313	0.69 (0.441–1.079)	0.104	0.72 (0.458-1.132)	0.155
PNI ≥ 48	Ref.		Ref.		Ref.		Ref.	
PNI < 48	1.79 (1.394–2.299)	<0.001	1.577 (1.218–2.042)	0.001	1.332 (1.013–1.751)	0.04	1.338 (1.016-1.763)	0.038

Multivariable analysis model 1: adjusting for gender, congestive heart failure and chronic pulmonary disease. Multivariable analysis model 2: adjusting for variables in model 1 as well as SOFA score, MBP, preoperative hematocrit, and BUN. Multivariable analysis model 3: adjusting for variables in model 2 as well as postoperative creatinine and BUN. GNRI, geriatric nutritional risk index; PNI, prognostic nutritional index; AKI, acute kidney injury; CABG, coronary artery bypass grafting; OR, odds ratio, Ref, reference.

**Table 3.** Performance of models with GNRI and PNI to predict the postoperative moderate to severe AKI in older patients undergoing CABG.

	AUC		NRI		IDI	
	AUC (95%)	*P*	index (95%)	*P*	index (95%)	*P*
Nutritional indices as continuous variables						
model1	0.607 (0.574 − 0.64)					
model1 + GNRI	0.635 (0.601 − 0.67)	0.009	0.027 (-0.012 − 0.067)	0.176	0.017 (0.009 − 0.024)	<0.001
model1 + PNI	0.64 (0.606 − 0.674)	0.004	0.024 (-0.019 − 0.067)	0.266	0.024 (0.015 − 0.034)	<0.001
model1 + GNRI+PNI	0.708 (0.676 − 0.739)	<0.001	0.097 (0.039 − 0.155)	0.001	0.083 (0.067 − 0.100)	<0.001
model2	0.659 (0.626 − 0.693)					
model2 + GNRI	0.685 (0.653 − 0.718)	0.007	0.059 (0.009 − 0.110)	0.021	0.026 (0.016 − 0.035)	<0.001
model2 + PNI	0.674 (0.641 − 0.706)	0.050	−0.0134 (-0.056 − 0.029)	0.534	0.016 (0.008 − 0.024)	<0.001
model2 + GNRI+PNI	0.731 (0.70 − 0.761)	<0.001	0.0953 (0.036 − 0.154)	0.002	0.075 (0.059 − 0.091)	<0.001
model3	0.674 (0.642 − 0.707)					
model3 + GNRI	0.694 (0.661 − 0.726)	0.020	0.0082 (-0.034 − 0.050)	0.701	0.020 (0.012 − 0.029)	<0.001
model3 + PNI	0.688 (0.656 − 0.721)	0.042	0.0227 (-0.019 − 0.064)	0.283	0.016 (0.008 − 0.024)	<0.001
model3 + GNRI+PNI	0.737 (0.706 − 0.767)	<0.001	0.1042 (0.049 − 0.160)	0.000	0.067 (0.052 − 0.082)	<0.001
Nutritional indices as category variables						
model1	0.607 (0.574 − 0.64)					
model1 + GNRI	0.611 (0.577 − 0.644)	0.143	−0.007 (-0.015 − 0)	0.045	0.001 (-0.001 − 0.003)	0.320
model1 + PNI	0.622 (0.588 − 0.656)	0.141	−0.006 (-0.048 − 0.035)	0.770	0.011 (0.005 − 0.018)	0.001
model1 + GNRI+PNI	0.627 (0.593 − 0.661)	0.057	0 (-0.039 − 0.039)	0.984	0.015 (0.007 − 0.022)	<0.001
model2	0.659 (0.626 − 0.693)					
model2 + GNRI	0.662 (0.628 − 0.695)	0.512	−0.006 (-0.027 − 0.015)	0.572	0.002 (-0.001–0.005)	0.158
model2 + PNI	0.663 (0.629 − 0.696)	0.486	−0.036 (-0.075 − 0.003)	0.069	0.004 (0 − 0.008)	0.049
model2 + GNRI+PNI	0.666 (0.633 − 0.699)	0.270	−0.018 (-0.061 − 0.024)	0.395	0.008 (0.002 − 0.013)	0.006
model3	0.674 (0.642 − 0.707)					
model3 + GNRI	0.676 (0.643 − 0.708)	0.655	−0.005 (-0.028 − 0.018)	0.680	0.002 (-0.001 − 0.004)	0.258
model3 + PNI	0.678 (0.645 − 0.71)	0.444	−0.023 (-0.059 − 0.014)	0.221	0.004 (0 − 0.008)	0.043
model3 + GNRI+PNI	0.68 (0.647 − 0.712)	0.332	−0.005 (-0.045 − 0.034)	0.792	0.007 (0.002 − 0.012)	0.009

Multivariable analysis model 1: adjusting for gender, congestive heart failure and chronic pulmonary disease. Multivariable analysis model 2: adjusting for variables in model 1 as well as SOFA score, MBP, preoperative hematocrit and BUN. Multivariable analysis model 3: adjusting for variables in model 2 as well as postoperative creatinine and BUN. GNRI, geriatric nutritional risk index; PNI, prognostic nutritional index; AKI, acute kidney injury; CABG, coronary artery bypass grafting; AUC, area under curve; NRI, Net reclassification improvement; IDI, Integrated discrimination improvement.

### 3.3. The association between nutrition scores and moderate-to-severe AKI

Covariables that were associated with moderate-to-severe AKI following CABG surgery in the adjusted model were screened (Table S4).

**Figure 3.**
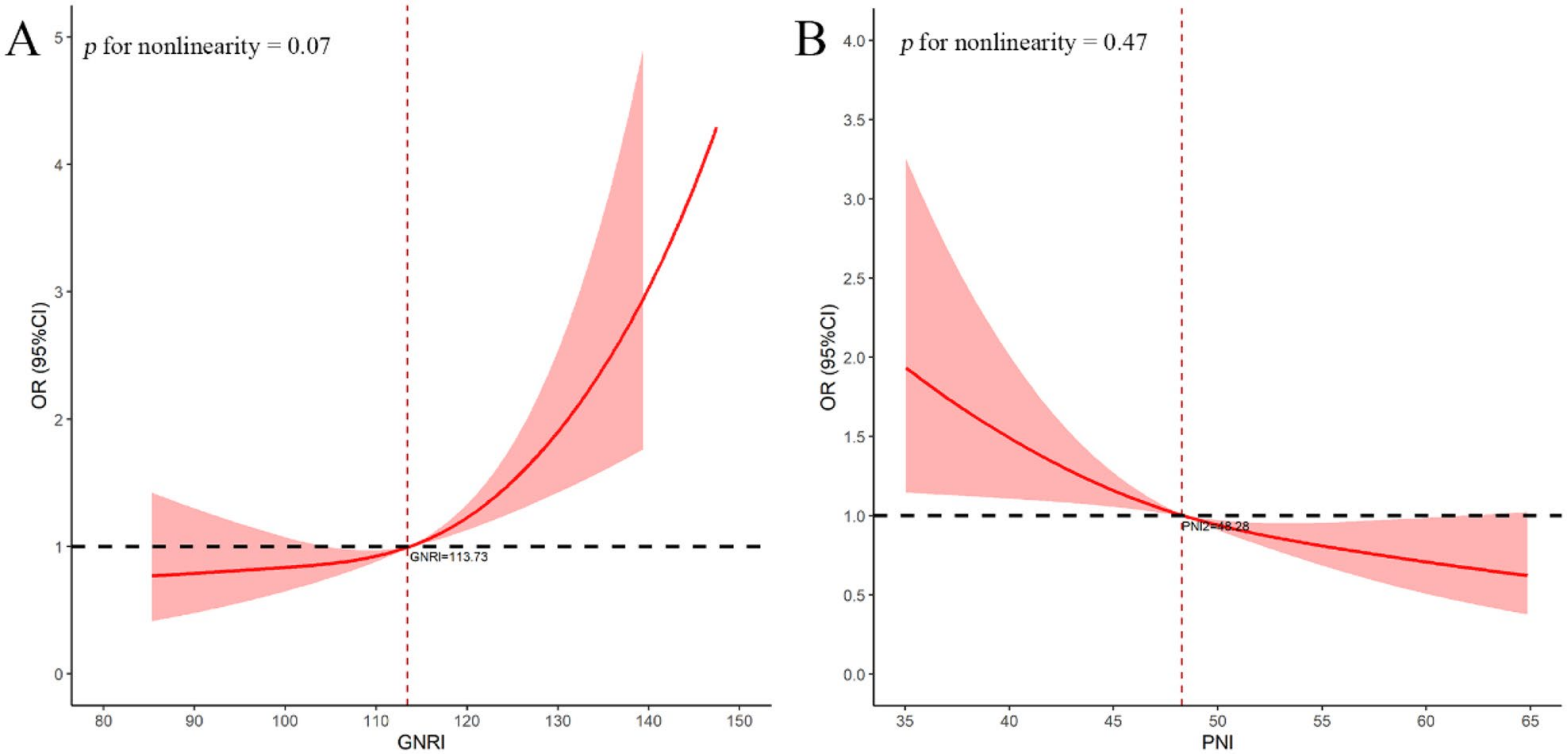
Dose–response relationships between GNRI (A) and PNI (B) and moderate to severe AKI in older patients undergoing CABG. The red bold line illustrates the or, while the shaded area indicates the 95% CI. They were adjusted for gender, congestive heart failure, chronic pulmonary disease, SOFA, MBP, preoperative hematocrit, preoperative BUN, postoperative creatinine and postoperative BUN. Vertical dotted lines reflect the minimal threshold for the beneficial association with estimated or = 1. Abbreviations: GNRI, geriatric nutritional risk index; PNI, prognostic nutritional index; AKI, acute kidney injury; CABG, coronary-artery-bypass-grafting. OR, odds ratio; CI, confidence interval; SOFA, sequential organ failure assessment; MBP, mean blood pressure; BUN, blood urea nitrogen.

GNRI and PNI were firstly incorporated as continuous variables for logistic regression, revealing that they were significantly associated with the odds ratio of moderate to severe AKI, ranging from the univariate model to different multivariate models (all *p* < 0.05; Table 2). After adjusting the screened variable by multivariable analysis, the RCS was utilized to illustrate the association between them. A linear association was indicated between nutrition scores and the OR of moderate to severe AKI (*p* for nonlinearity > 0.05). Specifically, the OR of moderate-to-severe AKI increased as GNRI score elevated. Regarding PNI, there was a significant decline in the OR of moderate to severe AKI with increasing PNI. The optimal cutoff points of GNRI and PNI were 113.73 and 48.28, respectively (Figure 3).

**Figure 4.**
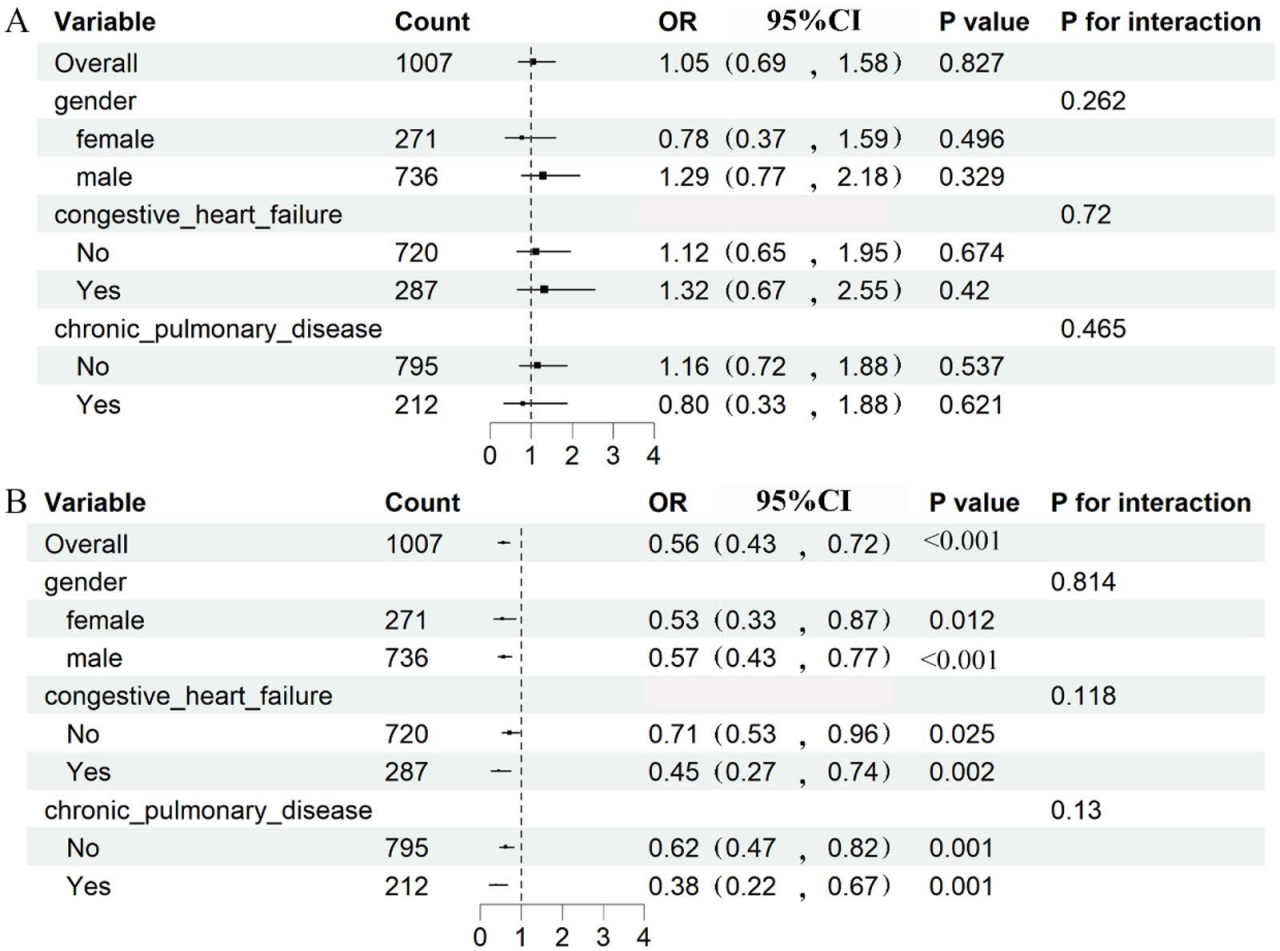
Subgroup analysis for the association of malnutrition regrading as categorical variables and moderate to severe AKI in different subgroups. OR of GNRI (a), and PNI (B) were adjusted by the variables that are stratified by itself only. OR, odds ratio; GNRI, geriatric nutritional risk index; PNI, prognostic nutritional index.

GNRI and PNI scores were also distinguished into categorical variables to further study the association between nutrition risk and moderate-to-severe AKI after CABG surgery *via* logistic regression. As the results demonstrated, there was no significant association between the nutrition risk calculated by GNRI and the incidence of moderate to severe AKI (*p* > 0.05). In contrast, the PNI-defined nutrition risk was significantly associated with the elevated risk of moderate to severe AKI (*p* < 0.05; Table 2). To be specific, the odds ratio of moderate-to-severe AKI was more than 1.338(1.016–1.763) times greater than those without nutrition risk. Assessing the association between the malnutrition and the risk of moderate-to-severe AKI among geriatric patients underwent CABG surgery, while taking into categorical variances such as gender, congestive heart failure, and chronic pulmonary disease. The results demonstrated that there were no significant interaction effects across different subgroups in the subgroup analysis (Figure 4).

### 3.4. Incremental value of nutrition scores and comparative analysis

We firstly consider the GNRI and PNI scores as continuous data. When adding them separately to three base models, the results demonstrated that the addition of GNRI or PNI to the base model 1 significantly elevated AUC and IDI, but the NRI weren’t significantly improved (Table 3 and Figure S1A). The AUC and IDI of the base model 2 elevated significantly when the two indices were included, but only GNRI significantly improved NRI (Table 3 and Figure S1B). Similarly, adding the two indices to the base model 3 significantly improved AUC and IDI without significant NRI changes (Table 3 and Figure S1C). When adding two indicators simultaneously to three base models, the results indicated that it can significantly improve the AUCs, NRIs, and IDIs (Table 3 and Figure S1). When the two nutritional indicators were compared, the results revealed that their AUCs were similar and that their differences of NRI and IDI were not statistically significant in different models (Table S5 and Figure S1).

Next, we calculated the incremental predictive value of the GNRI and PNI regarding as categorical variables. When adding them respectively to three base models, the addition of everyone to the base model 1 did not significantly improve the AUC and NRI, but PNI merely leads to a significant increment in terms of IDI (Table 3 and Figure S2A). This outcome was consistent with model 2 and 3 (Table 3 and Figure S2B–C). When adding two indicators to three base models at the same time, the results showed that it can significantly improve the IDIs (Table 3 and Figure S2). When the two nutritional indicators were compared, the results indicated that they have no significant differences in terms of AUCs, NRIs, and IDIs (Table S5 and Figure S2).

In addition, calibration curves also demonstrated good performance between model-predicted moderate to severe AKI risk and the actual moderate to severe AKI probability in three models (Figure S3–S4). Finally, multivariable models integrating different nutritional scores showed higher net benefits at decision thresholds between 30% and 80%, according to DCA (Figure S5–S6).

## 4. Discussion

### 4.1. Main findings

In the retrospective study, the results revealed that preoperative nutritional status, a potential independent predictor, was significantly associated with moderate-to-severe AKI following CABG surgery in elderly patients. Additionally, we discovered that integrating nutritional indices into the basic predictive models enhanced their predictive performance. There was no difference in the incremental prediction significance of the two indicators.

### 4.2. Possible explanations for findings

The precise mechanisms by which malnutrition contributes to AKI remain to be fully determined. However, the pronounced catabolism, a characteristic of critical illness following CABG surgery, provides a partial explanation [33]. Excessive catabolism can damage renal and adrenal cells through increased inflammatory responses and oxidative stress. This not only directly precipitates AKI but also disrupts systemic glucose and lipid metabolism and induces insulin resistance by impacting adrenal function, thereby exacerbating malnutrition [34,35]. Furthermore, heightened catabolism leads to reduced serum albumin levels and plasma colloid osmotic pressure, causing intravascular water to shift to peripheral tissues. This shift results in a decreased effective circulating blood volume, subsequently impairing renal perfusion and precipitating AKI. Additionally, the inflammatory response can induce vasoconstriction, further leading to renal hypoperfusion [33,36].

Albumin has been conventionally regarded as an indicator of patients’ nutritional status. However, this perspective is increasingly challenged by contemporary studies [37,38]. Recent studies suggest that albumin characterizes inflammatory processes rather than nutritional status or protein-energy malnutrition. Inflammatory mediators significantly suppress the synthesis of albumin and induce the redistribution of serum proteins by increasing capillary permeability as part of the inflammatory response [39,40]. Therefore, albumin more accurately predicts the risk of adverse outcomes in patients, diverging from its traditional associated with malnutrition. In addition, lymphocytes are the important laboratory results to assess nutritional status. Due to the characteristics of immunosuppression in the critical illness, lymphocytes played an integral part in the occurrence, development and recovery of AKI [34,41]. Many clinical studies indicated that decreased preoperative lymphocyte counts were associated with incremental risk of postoperative AKI and in-hospital mortality [42,43].

The widely used cutoff values of GNRI to assess malnutrition was established by Bouillanne et al. [27]. However, the cutoff values may vary across different patient populations and diseases. For instance, the optimal GNRI cutoff to predict overall survival among older patients with diffuse large B-cell lymphoma was 106.26 [29], whereas for elderly patients who received curative resection for colorectal cancer, the cutoff was 101.1 [44]. Furthermore, the ideal GNRI cutoffs for predicting mortality among adult hemodialysis patients varied according to age and gender [45]. In addition, the above studies primarily examined the relationship between GNRI and mortality, while this study concentrates on the association between GNRI and the incidence of moderate to severe AKI. In our findings, the optimal GNRI cutoff value for predicting moderate to severe AKI is determined to be 113.42 by RCS. This may be the reason why there was no significant association between malnutrition and moderate-to-severe AKI when 98 was regarded as the cutoff value. We also find that the OR for moderate-to-severe AKI increases as the GNRI value raises, which is consistent with previous studies [46,47]. This underscores the significance of considering the GNRI cutoff variability when assessing risk and prognosis across different medical conditions and patients.

The GNRI is largely determined by actual body weight. Increased GNRI may have elevated the risk of moderate to severe AKI in patients underwent the CABG surgery. The pathophysiological mechanism behind this may be associated with obesity-related renal dysfunction. Several possible mechanisms have been suggested to explain. On the one hand, obesity directly contributes to kidney damage by altering hemodynamics. It leads to a state of ‘hyperperfusion, hyperpressure, and hyperfiltration’ in the glomeruli, which increases the tension in the glomerular capillary walls and compromises the glomerular filtration barrier. This alteration causes the leakage of proteins, which are toxic to mesangial cells and podocytes in the kidneys, thereby exacerbating tubulointerstitial damage and accelerating glomerulosclerosis, which ultimately leads to kidney injury [48,49]. On the other hand, obesity is commonly associated with metabolic dysregulation, characterized by insulin resistance and a low-grade inflammatory state. These conditions collectively exacerbate renal damage by promoting systemic and renal-specific inflammatory pathways [50,51]. Such mechanisms underscore the intricate link between obesity and kidney health, underscoring the significance of managing nutritional status by regulating obesity to mitigate its adverse effects on renal function.

### 4.3. Clinical implications

This study has underlying clinical impact, especially for elderly patients who will undergo the CABG surgery. First, it underscores the significant association between preoperative nutritional condition and postoperative moderate to severe AKI. Previous studies have indicated that most patients after cardiac surgery exist late nutritional support and inadequate feeding [52]. Screening and managing of malnutrition were essential parts of the surgical enhanced recovery program. Utilizing GNRI and PNI facilitates earlier and more precise assessment of nutritional status, providing new insights for perioperative nutritional intervention. Second, extensive studies have indicated that preoperative malnutrition is a major risk factor contributing to postoperative AKI [19,20,53]. This study further confirms that the predictive performance of models incorporating nutritional scores is superior. This aids in prediction of moderate-to-severe AKI in advance, providing novel theoretical support for comprehensive management of AKI. The guideline has also recommended to evaluate and manage preoperatively malnutrition before cardiac surgery to decrease the incidence of postoperative complications [54]. Third, the specialized nutritional interventions were required for patients with severe AKI to correct electrolyte disorders and negative nitrogen balance [55,56]. Although studies have suggested that the timing of parenteral nutrition intervention may affect the time required for renal function recovery [57], literature regarding nutritional intervention’s safety and efficacy among AKI patients is limited and a suitable approach for enhancing nutritional status remains for patients with severe AKI was unclear. There was no standard strategy for improving nutritional status because every patient’s nutritional condition and comorbidities vary. Further studies are needed to explore the effects of different nutritional managements on renal function recovery in patients with severe AKI, especially for older patients after CABG surgery.

### 4.4. Strengths and limitations

Our study possesses several notable strengths. Primarily, it concentrated on the high-risk individuals: older patients undergoing CABG surgery. Additionally, our research evaluated the association between preoperative nutritional status and postoperative moderate to severe AKI in the elderly underwent CABG surgery, as well as the predictive performance of various nutritional indicators for moderate to severe AKI through comprehensive approaches. Lastly, we made a comparative analysis to have a better understanding of the predictive efficacy of GNRI and PNI, which contributed valuable insights into their relative performance.

The study is not without limitations. First, being a single-center, retrospective, and observational study, it didn’t establish a causal link between malnutrition and moderate to severe AKI, which multi-center studies are necessary to further validation. Second, the absence of CONUT, MUST, and NRS-2002 scores limited the generalizability of our findings, as these indices are more commonly employed in clinical settings. Third, the nutritional indices used may not capture the entire spectrum of malnutrition, omitting aspects like vitamin deficiencies [58,59], skeletal muscle index [60,61], and frailty [19], which are known to be associated with AKI. Fourth, cardiopulmonary bypass is an independent risk factor for postoperative AKI. However, the relevant information was lacked in the MIMIC database, and we cannot further explore the predictive performance of preoperative nutritional status in off-pump or on-pump patients. Last, the exclusion of many patients due to incomplete preoperative nutritional information or AKI data could introduce selection bias. Furthermore, potential influences on GNRI and PNI indices from internal factors such as hormonal fluctuations, inflammation, and changes in the circulating blood volume were not accounted for, possibly leading to another selection bias.

## 5. Conclusion

This study reported malnutrition status, evaluated by GNRI and PNI, was independently associated with the incidence of moderate to severe AKI in geriatrics following CABG surgery. Adding two indicators to base models of moderate to severe AKI made the predictive performance better. Collectively, these results underscored the potential value of GNRI and PNI monitoring in forecasting moderate to severe AKI following CABG, thereby paving the way for future research on their clinical utility and implementation.

## Supplementary Material

supplementary material.docx

## Data Availability

Data are available in a public, open access repository. The datasets utilized during the current study are available in the MIMIC- IV repository (https://physionet.org/content/mimiciv/2.2/).

## References

[1] Garg AX, Devereaux PJ, Yusuf S, et al. Kidney function after off-pump or on-pump coronary artery bypass graft surgery: a randomized clinical trial. JAMA. 2014;311(21):2191–2198. doi: 10.1001/jama.2014.4952. 24886787

[2] Lamy A, Devereaux PJ, Prabhakaran D, et al. Off-pump or on-pump coronary-artery bypass grafting at 30 days. N Engl J Med. 2012;366(16):1489–1497. doi: 10.1056/NEJMoa1200388. 22449296

[3] Cheungpasitporn W, Thongprayoon C, Kittanamongkolchai W, et al. Comparison of renal outcomes in off-pump versus on-pump coronary artery bypass grafting: a systematic review and meta-analysis of randomized controlled trials. Nephrology (Carlton). 2015;20(10):727–735. doi: 10.1111/nep.12506. 25968971

[4] Diegeler A, Börgermann J, Kappert U, et al. Off-pump versus on-pump coronary-artery bypass grafting in elderly patients. N Engl J Med. 2013;368(13):1189–1198. doi: 10.1056/NEJMoa1211666. 23477657

[5] Cheruku SR, Raphael J, Neyra JA, et al. Acute kidney injury after cardiac surgery: prediction, prevention, and management. Anesthesiology. 2023;139(6):880–898. doi: 10.1097/ALN.0000000000004734. 37812758 PMC10841304

[6] Corredor C, Thomson R, Al-Subaie N. Long-term consequences of acute kidney injury after cardiac surgery: a systematic review and meta-analysis. J Cardiothorac Vasc Anesth. 2016; 30(1):69–75. doi: 10.1053/j.jvca.2015.07.013. 26482483

[7] Gallagher M, Cass A, Bellomo R, et al. Long-term survival and dialysis dependency following acute kidney injury in intensive care: extended follow-up of a randomized controlled trial. PLoS Med. 2014;11(2):e1001601. doi: 10.1371/journal.pmed.1001601. 24523666 PMC3921111

[8] Hobson CE, Yavas S, Segal MS, et al. Acute kidney injury is associated with increased long-term mortality after cardiothoracic surgery. Circulation. 2009;119(18):2444–2453. doi: 10.1161/CIRCULATIONAHA.108.800011. 19398670

[9] Chawla LS, Bellomo R, Bihorac A, et al. Acute kidney disease and renal recovery: consensus report of the Acute Disease Quality Initiative (ADQI) 16 Workgroup. Nat Rev Nephrol. 2017;13(4):241–257. doi: 10.1038/nrneph.2017.2. 28239173

[10] Wang Y, Bellomo R. Cardiac surgery-associated acute kidney injury: risk factors, pathophysiology and treatment. Nat Rev Nephrol. 2017;13(11):697–711. doi: 10.1038/nrneph.2017.119. 28869251

[11] Besora-Moreno M, Llauradó E, Tarro L, et al. Social and economic factors and malnutrition or the risk of malnutrition in the elderly: a systematic review and meta-analysis of observational studies. Nutrients. 2020;12(3):737. doi: 10.3390/nu12030737. 32168827 PMC7146387

[12] Dent E, Wright ORL, Woo J, et al. Malnutrition in older adults. Lancet. 2023;401(10380):951–966. doi: 10.1016/S0140-6736(22)02612-5. 36716756

[13] Serón-Arbeloa C, Labarta-Monzón L, Puzo-Foncillas J, et al. Malnutrition screening and assessment. Nutrients. 2022;14(12):2392. doi: 10.3390/nu14122392. 35745121 PMC9228435

[14] Wei F, Cheng H, He R, et al. Geriatric nutritional risk index independently predicts delirium in older patients in intensive care units: a multicenter cohort study. Arch Gerontol Geriatr. 2024;118:105288. doi: 10.1016/j.archger.2023.105288. 38056103

[15] Xiong J, Yu Z, Huang Y, et al. Geriatric nutritional risk index and risk of mortality in critically ill patients with acute kidney injury: a multicenter cohort study. J Ren Nutr. 2023;33(5):639–648. doi: 10.1053/j.jrn.2023.06.004. 37302721

[16] Abd Aziz NAS, Mohd Fahmi Teng NI, Kamarul Zaman M. Geriatric Nutrition Risk Index is comparable to the mini nutritional assessment for assessing nutritional status in elderly hospitalized patients. Clin Nutr ESPEN. 2019;29:77–85. doi: 10.1016/j.clnesp.2018.12.002. 30661705

[17] Baldemir R, Cirik M. Practical parameters that can be used for nutritional assessment in patients hospitalized in the intensive care unit with the diagnosis of chronic obstructive pulmonary disease: prognostic nutritional index, neutrophil-to-lymphocyte, platelet-to-lymphocyte, and lymphocyte-to-monocyte ratio. Medicine (Baltimore).). 2022;101(24):e29433. doi: 10.1097/MD.0000000000029433. 35713452 PMC9276300

[18] Zhang X, Wang Y, Xu M, et al. The malnutrition in AECOPD and its association with unfavorable outcomes by comparing PNI, GNRI with the GLIM criteria: a retrospective cohort study. Front Nutr. 2024;11:1365462. doi: 10.3389/fnut.2024.1365462. 39183991 PMC11341410

[19] Aykut A, Salman N. Poor nutritional status and frailty associated with acute kidney injury after cardiac surgery: a retrospective observational study. J Card Surg. 2022;37(12):4755–4761. doi: 10.1111/jocs.17134. 36352787

[20] Dolapoglu A, Avci E, Kiris T, et al. The predictive value of the prognostic nutritional index for postoperative acute kidney injury in patients undergoing on-pump coronary bypass surgery. J Cardiothorac Surg. 2019;14(1):74. doi: 10.1186/s13019-019-0898-7. 30971264 PMC6458745

[21] Usta S, Engin M. Investigation of the effects of preoperative nutritional status scores on renal injury after cardiac surgery in elderly patients. Eur Rev Med Pharmacol Sci. 2022;26(24):9345–9352. doi: 10.26355/eurrev_202212_30685. 36591843

[22] Tóth K, Szabó A, Nagy Á, et al. Preoperative nutritional state is associated with mid- and long-term mortality after cardiac surgery. Ann Palliat Med. 2021;10(11):11333–11347. doi: 10.21037/apm-21-1015. 34670385

[23] Gucu A, Ozluk OA, Sunbul SA, et al. Prognostic nutritional index as a marker of mortality: an observational cohort study of patients undergoing cardiac surgery. Rev Cardiovasc Med. 2021;22(2):499–503. doi: 10.31083/j.rcm2202057. 34258918

[24] Cui X, Shen P, Jin L, et al. Preoperative prognostic nutritional index is an independent indicator for perioperative prognosis in coronary artery bypass grafting patients. Nutrition. 2023;116:112215. doi: 10.1016/j.nut.2023.112215. 37820569

[25] KDIGO 2024 Clinical practice guideline for the evaluation and management of chronic kidney disease. Kidney Int. 2024; 105(4):S117–s314. doi: 10.1016/j.kint.2023.10.018. 38490803

[26] Lameire NH, Levin A, Kellum JA, et al. Harmonizing acute and chronic kidney disease definition and classification: report of a Kidney Disease: improving Global Outcomes (KDIGO) Consensus Conference. Kidney Int. 2021;100(3):516–526. doi: 10.1016/j.kint.2021.06.028. 34252450

[27] Bouillanne O, Morineau G, Dupont C, et al. Geriatric Nutritional Risk Index: a new index for evaluating at-risk elderly medical patients. Am J Clin Nutr. 2005;82(4):777–783. doi: 10.1093/ajcn/82.4.777. 16210706

[28] Hayashi J, Uchida T, Ri S, et al. Clinical significance of the prognostic nutritional index in patients undergoing cardiovascular surgery. Gen Thorac Cardiovasc Surg. 2020;68(8):774–779. doi: 10.1007/s11748-020-01300-x. 32088837

[29] Yan D, Shen Z, Zhang S, et al. Prognostic values of geriatric nutritional risk index (GNRI) and prognostic nutritional index (PNI) in elderly patients with diffuse large B-cell lymphoma. J Cancer. 2021;12(23):7010–7017. doi: 10.7150/jca.62340. 34729103 PMC8558670

[30] Groenwold RHH, Dekkers OM. Subgroup analyses in clinical research: too tempting? Eur J Endocrinol. 2023; 189(2):E1–E3. doi: 10.1093/ejendo/lvad089. 37527541

[31] Riley RD, Ensor J, Snell KIE, et al. Calculating the sample size required for developing a clinical prediction model. BMJ. 2020;368:m441. doi: 10.1136/bmj.m441. 32188600

[32] Birnie K, Verheyden V, Pagano D, et al. Predictive models for kidney disease: improving global outcomes (KDIGO) defined acute kidney injury in UK cardiac surgery. Crit Care. 2014;18(6):606. doi: 10.1186/s13054-014-0606-x. 25673427 PMC4258283

[33] Chadda KR, Puthucheary Z. Persistent inflammation, immunosuppression, and catabolism syndrome (PICS): a review of definitions, potential therapies, and research priorities. Br J Anaesth. 2024; 132(3):507–518. doi: 10.1016/j.bja.2023.11.052. 38177003 PMC10870139

[34] Singbartl K, Formeck CL, Kellum JA. Kidney-Immune System Crosstalk in AKI. Semin Nephrol. 2019;39(1):96–106. doi: 10.1016/j.semnephrol.2018.10.007. 30606411

[35] MacLaughlin HL, Friedman AN, Ikizler TA. Nutrition in kidney disease: core curriculum 2022. Am J Kidney Dis. 2022;79(3):437–449. doi: 10.1053/j.ajkd.2021.05.024. 34862042

[36] Zhang J, Luo W, Miao C, et al. Hypercatabolism and anti-catabolic therapies in the persistent inflammation, immunosuppression, and catabolism syndrome. Front Nutr. 2022;9:941097. doi: 10.3389/fnut.2022.941097. 35911117 PMC9326442

[37] Keller U. Nutritional laboratory markers in malnutrition. J Clin Med. 2019;8(6):775. doi: 10.3390/jcm8060775. PMC6616535 31159248

[38] Evans DC, Corkins MR, Malone A, et al. The use of visceral proteins as nutrition markers: an ASPEN Position Paper. Nutr Clin Pract. 2021;36(1):22–28. doi: 10.1002/ncp.10588. 33125793

[39] Sharma K, Mogensen KM, Robinson MK. Pathophysiology of critical illness and role of nutrition. Nutr Clin Pract. 2019;34(1):12–22. doi: 10.1002/ncp.10232. 30580456

[40] Li Z, Zhao H, Wang J. Metabolism and chronic inflammation: the links between chronic heart failure and comorbidities. Front Cardiovasc Med. 2021;8:650278. doi: 10.3389/fcvm.2021.650278. 34026868 PMC8131678

[41] Noel S, Lee K, Gharaie S, et al. Immune checkpoint molecule TIGIT regulates kidney T cell functions and contributes to AKI. J Am Soc Nephrol. 2023;34(5):755–771. doi: 10.1681/ASN.0000000000000063. 36747315 PMC10125646

[42] Jeon YH, Jeon Y, Jung HY, et al. Platelet-to-lymphocyte ratio and in-hospital mortality in patients with AKI receiving continuous kidney replacement therapy: a retrospective observational cohort study. Kidney Med. 2023;5(6):100642. doi: 10.1016/j.xkme.2023.100642. 37235040 PMC10205757

[43] Wei W, Huang X, Yang L, et al. Neutrophil-to-lymphocyte ratio as a prognostic marker of mortality and disease severity in septic acute kidney injury patients: a retrospective study. Int Immunopharmacol. 2023;116:109778. doi: 10.1016/j.intimp.2023.109778. 36738677

[44] Hayama T, Hashiguchi Y, Ozawa T, et al. The preoperative geriatric nutritional risk index (GNRI) is an independent prognostic factor in elderly patients underwent curative resection for colorectal cancer. Sci Rep. 2022;12(1):3682. doi: 10.1038/s41598-022-07540-6. 35256659 PMC8901671

[45] Kim DH, Lee YK, Park HC, et al. The geriatric nutritional risk index is an optimal evaluation parameter for predicting mortality in adult all ages hemodialysis patients: a Korean population-based study. Nutrients. 2023;15(17):3831. doi: 10.3390/nu15173831. PMC10490009 37686863

[46] Li D, Chen Z, He W, et al. The association between nutritional risk and contrast-induced acute kidney injury in patients undergoing coronary angiography: a cross-sectional study. Nutr J. 2022;21(1):56. doi: 10.1186/s12937-022-00810-z. 36114539 PMC9479352

[47] Sun R, Zhou Z, Li X, et al. Prognostic significance of preoperative nutritional status for postoperative acute kidney injury in older patients undergoing major abdominal surgery: a retrospective cohort study. Int J Surg. 2024;110(2):873–883. doi: 10.1097/JS9.0000000000000861. 37921644 PMC10871641

[48] Câmara NO, Iseki K, Kramer H, et al. Kidney disease and obesity: epidemiology, mechanisms and treatment. Nat Rev Nephrol. 2017;13(3):181–190. doi: 10.1038/nrneph.2016.191. 28090083

[49] Hall JE, do Carmo JM, da Silva AA, et al. Obesity-induced hypertension: interaction of neurohumoral and renal mechanisms. Circ Res. 2015;116(6):991–1006. doi: 10.1161/CIRCRESAHA.116.305697. 25767285 PMC4363087

[50] D’Agati VD, Chagnac A, de Vries AP, et al. Obesity-related glomerulopathy: clinical and pathologic characteristics and pathogenesis. Nat Rev Nephrol. 2016;12(8):453–471. doi: 10.1038/nrneph.2016.75. 27263398

[51] Bayliss G, Weinrauch LA, D’Elia JA. Pathophysiology of obesity-related renal dysfunction contributes to diabetic nephropathy. Curr Diab Rep. 2012;12(4):440–446. doi: 10.1007/s11892-012-0288-1. 22638939

[52] Mertes PM, Kindo M, Amour J, et al. Guidelines on enhanced recovery after cardiac surgery under cardiopulmonary bypass or off-pump. Anaesth Crit Care Pain Med. 2022;41(3):101059. doi: 10.1016/j.accpm.2022.101059. 35504126

[53] Wang XM, Deng YS, He B, et al. The serum anion gap is associated with the prognosis of coronary artery bypass grafting (CABG): analysis based on the MIMIC-IV database. Eur Rev Med Pharmacol Sci. 2023;27(7):2964–2970. 37070897 10.26355/eurrev_202304_31928

[54] Engelman DT, Ben Ali W, Williams JB, et al. Guidelines for perioperative care in cardiac surgery: enhanced recovery after surgery society recommendations. JAMA Surg. 2019;154(8):755–766. doi: 10.1001/jamasurg.2019.1153. 31054241

[55] Fiaccadori E, Regolisti G, Maggiore U. Specialized nutritional support interventions in critically ill patients on renal replacement therapy. Curr Opin Clin Nutr Metab Care. 2013; 16(2):217–224. doi: 10.1097/MCO.0b013e32835c20b0. 23242314

[56] Scheinkestel CD, Kar L, Marshall K, et al. Prospective randomized trial to assess caloric and protein needs of critically Ill, anuric, ventilated patients requiring continuous renal replacement therapy. Nutrition. 2003;19(11-12):909–916. doi: 10.1016/s0899-9007(03)00175-8. 14624937

[57] Casaer MP, Mesotten D, Hermans G, et al. Early versus late parenteral nutrition in critically ill adults. N Engl J Med. 2011;365(6):506–517. doi: 10.1056/NEJMoa1102662. 21714640

[58] Jiang S, Huang L, Zhang W, et al. Vitamin D/VDR in acute kidney injury: a potential therapeutic target. Curr Med Chem. 2021;28(19):3865–3876. doi: 10.2174/0929867327666201118155625. 33213307

[59] Zhang H, Jiang Y, Shi N, et al. Serum vitamin D levels and acute kidney injury: a systemic review and meta-analysis. Sci Rep. 2022;12(1):20365. doi: 10.1038/s41598-022-24560-4. 36437252 PMC9701671

[60] Sim JH, Kwon HM, Jun IG, et al. Association of skeletal muscle index with postoperative acute kidney injury in living donor hepatectomy: A retrospective single-centre cohort study. Liver Int. 2022;42(2):425–434. doi: 10.1111/liv.15109. 34817911

[61] Bang JY, Jun IG, Lee JB, et al. Impact of sarcopenia on acute kidney injury after infrarenal abdominal aortic aneurysm surgery: a propensity matching analysis. Nutrients. 2021;13(7):2212. doi: 10.3390/nu13072212. 34199110 PMC8308481

