# Supplementary material for: Prognostic value of preoperative nutritional status for postoperative moderate to severe acute kidney injury among older patients undergoing coronary artery bypass graft surgery: a retrospective study based on the MIMIC-IV database
Source: Ren Fail. 2024 Dec 1;46(2):2429683. doi: 10.1080/0886022X.2024.2429683 (PMC11613410; doi:10.1080/0886022X.2024.2429683)
Supplement: supplementary material.docx [file IRNF_A_2429683_SM7838.docx]

**Prognostic value of preoperative nutritional status for postoperative moderate to severe acute kidney injury among older patients undergoing coronary artery bypass graft surgery: a retrospective study based on the MIMIC-IV database**

**Supplementary material**

**Table S1.** Baseline characteristics of study participants in different stages.

**Table S2.** Characteristics of the included patients (stratified by GNRI).

**Table S3.** Characteristics of the included patients (stratified by PNI).

**Table S4.** Multivariate logistic regression analysis of moderate to severe AKI in older patients undergoing CABG.

**Table S5.** Comparative analysis of the discrimination of each nutritional index for postoperative modern to severe AKI.

**Figure S1-S2.** ROC of different models.

**Figure S3-S4.** Calibration curves of different models.

**Figure S5-S6.** Decision curve analysis.

**Table S1.** Baseline characteristics of study participants in different stages.

|  | No AKI | Mild AKI | Moderate AKI | Severe AKI | *p* value |
| --- | --- | --- | --- | --- | --- |
| n | 209 | 274 | 450 | 74 |  |
| Gender (Male) | 160 (76.6) | 211 (77.0) | 318 (70.7) | 47 (63.5) | 0.042 |
| Age (year) | 72.49 [68.14, 77.00] | 73.38 [69.75, 79.02] | 73.76 [69.06, 79.23] | 75.25 [70.87, 80.69] | 0.019 |
| BMI (kg/m2) | 26.75 [24.34, 30.13] | 28.31 [25.47, 32.00] | 29.95 [27.19, 33.46] | 31.13 [27.69, 35.65] | <0.001 |
| Height (cm) | 170.00 [163.00, 178.00] | 170.00 [165.00, 175.00] | 171.69 [164.62, 178.00] | 172.83 [163.25, 178.00] | 0.503 |
| Weight (kg) | 77.30 [70.35, 87.10] | 82.90 [72.98, 92.96] | 87.55 [77.06, 101.21] | 92.15 [77.00, 101.80] | <0.001 |
| Comorbid disease |  |  |  |  |  |
| Charlson comorbidity index | 4.00 [3.00, 6.00] | 5.00 [4.00, 6.00] | 5.00 [4.00, 7.00] | 7.00 [6.00, 9.00] | <0.001 |
| Congestive heart failure | 32 (15.3) | 66 (24.1) | 146 (32.4) | 43 (58.1) | <0.001 |
| Peripheral vascular disease | 32 (15.3) | 39 (14.2) | 70 (15.6) | 12 (16.2) | 0.959 |
| Hypertension | 133 (63.6) | 165 (60.2) | 267 (59.3) | 23 (31.1) | <0.001 |
| Chronic pulmonary disease | 33 (15.8) | 53 (19.3) | 103 (22.9) | 23 (31.1) | 0.025 |
| Diabetes | 77 (36.8) | 109 (39.8) | 203 (45.1) | 38 (51.4) | 0.066 |
| Chronic liver disease | 1 (0.5) | 3 (1.1) | 9 (2.0) | 3 (4.1) | 0.142 |
| Stroke | 22 (10.5) | 13 (4.7) | 36 (8.0) | 4 (5.4) | 0.093 |
| Chronic kidney disease | 34 (16.3) | 58 (21.2) | 92 (20.4) | 43 (58.1) | <0.001 |
| Cerebrovascular disease | 30 (14.4) | 33 (12.0) | 59 (13.1) | 9 (12.2) | 0.894 |
| GCS | 14.00 [14.00, 15.00] | 14.00 [13.00, 15.00] | 14.00 [12.25, 15.00] | 13.00 [9.25, 14.00] | <0.001 |
| SOFA | 5.00 [4.00, 7.00] | 6.00 [4.00, 7.00] | 6.00 [4.00, 8.00] | 9.00 [7.00, 12.00] | <0.001 |
| Heart rate (beat) | 82.00 [77.00, 87.00] | 82.00 [76.00, 87.00] | 81.00 [76.00, 87.00] | 81.00 [78.00, 88.00] | 0.961 |
| MBP (mmHg) | 74.00 [71.00, 77.00] | 73.00 [70.00, 77.00] | 72.00 [69.00, 76.00] | 71.00 [67.00, 74.00] | <0.001 |
| SPO_2_ (%) | 98.00 [97.00, 99.00] | 98.00 [97.00, 99.00] | 98.00 [97.00, 99.00] | 98.00 [97.00, 99.00] | 0.377 |
| IABP | 9 (4.3) | 11 (4.0) | 31 (6.9) | 12 (16.2) | 0.001 |
| Ventilator | 176 (84.2) | 238 (86.9) | 390 (86.7) | 68 (91.9) | 0.418 |
| Vasopressor | 179 (85.6) | 232 (84.7) | 399 (88.7) | 66 (89.2) | 0.381 |
| Preoperative laboratories |  |  |  |  |  |
| Albumin (mg/dL) | 4.10 [3.80-4.45] | 4.10 [3.80, 4.45] | 3.90 [3.60, 4.20] | 3.75 [3.40, 4.00] | <0.001 |
| Creatinine (mg/dL) | 1.00 [0.80, 1.20] | 1.00 [0.90, 1.20] | 1.00 [0.90, 1.20] | 1.55 [1.02, 2.78] | <0.001 |
| Hematocrit (%) | 39.20 [34.75-41.85] | 39.20 [34.75, 41.85] | 37.00 [32.40, 40.93] | 33.65 [29.48, 37.65] | <0.001 |
| BUN (mg/dL) | 19.00 [16.00, 24.00] | 20.00 [16.00, 26.00] | 19.00 [15.00, 26.00] | 29.50 [21.00, 41.75] | <0.001 |
| Sodium (mmol/L) | 139.00 [137.00, 141.00] | 139.00 [137.25, 141.00] | 139.00 [137.00, 141.00] | 139.00 [137.00, 141.00] | 0.763 |
| Potassium (mmol/L) | 4.20 [4.00, 4.50] | 4.20 [4.00, 4.50] | 4.20 [3.90, 4.40] | 4.30 [4.00, 4.68] | 0.192 |
| Bicarbonate (mmol/L) | 26.00 [25.00, 28.00] | 26.00 [24.00, 28.00] | 26.00 [24.00, 28.00] | 26.00 [24.00, 28.00] | 0.346 |
| Lymphocyte (103/μL) | 1.73 [1.30, 2.25] | 1.64 [1.28, 2.14] | 1.60 [1.23, 2.05] | 1.42 [1.05, 1.92] | 0.001 |
| GNRI | 111.96 (10.99) | 113.44 (11.79) | 115.84 (13.16) | 114.32 (14.63) | 0.003 |
| GNRI≤98 | 20 (9.6) | 29 (10.6) | 39 (8.7) | 12 (16.2) | 0.237 |
| PNI | 50.24 [46.61, 54.05] | 48.86 [44.77, 53.88] | 47.80 [43.28, 51.88] | 44.27 [41.11, 47.73] | <0.001 |
| PNI < 48 | 75 (35.9) | 119 (43.4) | 229 (50.1) | 57 (87.0) | <0.001 |
| Postoperative laboratories |  |  |  |  |  |
| Creatinine (mg/dL) | 0.80 [0.70, 1.00] | 0.90 [0.70, 1.00] | 0.90 [0.70, 1.10] | 1.35 [0.92, 2.18] | <0.001 |
| BUN (mg /dL) | 16.00 [13.00, 19.00] | 17.00 [13.25, 21.00] | 17.00 [14.00, 21.00] | 25.50 [19.00, 37.00] | <0.001 |
| Sodium (mmol/L) | 139.00 [137.00, 141.00] | 139.00 [137.00, 141.00] | 139.00 [137.00, 140.00] | 139.00 [137.00, 141.00] | 0.29 |
| Potassium (mmol/L) | 4.20 [3.90, 4.50] | 4.30 [3.90, 4.60] | 4.30 [3.92, 4.60] | 4.50 [4.12, 5.07] | <0.001 |
| Bicarbonate (mmol/L) | 23.00 [22.00-24.00] | 23.00 [22.00, 24.00] | 23.00 [21.00, 24.00] | 22.00 [20.00, 23.25] | <0.001 |
| Time interval (h) | 3.41 [2.48, 4.80] | 3.38 [2.50, 4.75] | 3.50 [2.44, 4.89] | 4.00 [2.88, 5.63] | 0.16 |
| Outcomes |  |  |  |  |  |
| LOS in ICU (day) | 1.26 [1.12, 1.48] | 1.66 [1.24, 2.55] | 2.22 [1.33, 3.58] | 5.11 [3.30, 9.28] | <0.001 |
| LOS in hospital (day) | 6.82 [5.25, 9.27] | 7.44 [5.33, 10.16] | 8.81 [6.19, 11.83] | 13.39 [9.85, 20.01] | <0.001 |
| Hospital mortality (%) | 2 (1.0) | 1 (0.4) | 2 (0.4) | 8 (10.8) | <0.001 |
| Mortality 30‐day (%) | 2 (1.0) | 3 (1.1) | 4 (0.9) | 7 (9.5) | <0.001 |
| Mortality 90‐day (%) | 5 (2.4) | 7 (2.6) | 12 (2.7) | 14 (18.9) | <0.001 |

Values are expressed as median (interquartile range) or number of patients (%). AKI, acute kidney injury; BMI, body mass index; GCS, Glasgow coma scale; SOFA, sequential organ failure assessment; SPO_2_, oxyhemoglobin saturation by pulse oximetry; IABP, intra-aortic balloon pump; GNRI, geriatric nutritional risk index; PNI, prognostic nutritional index; BUN, blood urea nitrogen; LOS, length of stay.

**Table S2.** Characteristics of the included patients (stratified by GNRI).

|  | Malnutrition (≤98) | Normal (>98) | *p* value |
| --- | --- | --- | --- |
| n | 100 | 907 |  |
| Moderate to severe AKI | 51 (51.0) | 473 (52.1) | 0.237 |
| Gender (Male) | 64 (64.0) | 672 (74.1) | 0.041 |
| Age (year) | 77.07 [71.05, 82.84] | 73.21 [69.01, 78.67] | <0.001 |
| BMI (kg/m2) | 23.63 [21.95, 24.98] | 29.52 [26.65, 32.96] | <0.001 |
| Height (cm) | 168.00 [163.00, 175.00] | 170.30 [165.00, 178.00] | 0.13 |
| Weight (kg) | 68.05 [60.14, 74.78] | 86.10 [76.60, 97.85] | <0.001 |
| Comorbid disease |  |  |  |
| Charlson comorbidity index | 6.00 [4.00, 8.00] | 5.00 [4.00, 7.00] | 0.005 |
| Congestive heart failure | 43 (43.0) | 244 (26.9) | 0.001 |
| Peripheral vascular disease | 13 (13.0) | 140 (15.4) | 0.619 |
| Hypertension | 44 (44.0) | 544 (60.0) | 0.003 |
| Chronic pulmonary disease | 25 (25.0) | 187 (20.6) | 0.373 |
| Diabetes | 27 (27.0) | 400 (44.1) | 0.001 |
| Chronic liver disease | 3 (3.0) | 13 (1.4) | 0.443 |
| Stroke | 6 (6.0) | 69 (7.6) | 0.704 |
| Chronic kidney disease | 31 (31.0) | 196 (21.6) | 0.045 |
| Cerebrovascular disease | 14 (14.0) | 117 (12.9) | 0.878 |
| GCS | 14.00 [8.00, 15.00] | 14.00 [13.00, 15.00] | 0.015 |
| SOFA | 6.00 [4.00, 9.00] | 6.00 [4.00, 8.00] | 0.044 |
| Heart rate (beat) | 82.00 [77.00, 88.00] | 82.00 [76.00, 87.00] | 0.425 |
| MBP (mmHg) | 73.00 [68.00, 76.00] | 73.00 [69.00, 76.00] | 0.592 |
| SPO_2_ (%) | 98.00 [97.00, 99.00] | 98.00 [97.00, 99.00] | 0.01 |
| IABP | 12 (12.0) | 51 (5.6) | 0.023 |
| Ventilator | 88 (88.0) | 784 (86.4) | 0.779 |
| Vasopressor | 89 (89.0) | 787 (86.8) | 0.636 |
| Preoperative laboratories |  |  |  |
| Albumin (mg/dL) | 3.30 [3.00, 3.50] | 4.00 [3.70, 4.30] | <0.001 |
| Creatinine (mg/dL) | 1.00 [0.80, 1.40] | 1.00 [0.90, 1.20] | 0.883 |
| Hematocrit (%) | 32.20 [29.33, 38.25] | 37.90 [34.00, 41.40] | <0.001 |
| BUN (mg/dL) | 21.00 [17.75, 29.25] | 20.00 [16.00, 26.00] | 0.019 |
| Sodium (mmol/L) | 138.00 [136.00, 140.00] | 139.00 [137.00, 141.00] | 0.003 |
| Potassium (mmol/L) | 4.10 [3.90, 4.40] | 4.20 [4.00, 4.50] | 0.082 |
| Bicarbonate (mmol/L) | 25.00 [23.00, 27.00] | 26.00 [24.00, 28.00] | 0.001 |
| Lymphocyte (103/μL) | 1.45 [1.08, 1.93] | 1.64 [1.25, 2.12] | 0.05 |
| GNRI | 92.83 (4.86) | 116.63 (10.79) | <0.001 |
| PNI | 40.32 [37.05, 45.11] | 49.03 [44.96, 53.09] | <0.001 |
| Postoperative laboratories |  |  |  |
| Creatinine (mg/dL) | 0.90 [0.70, 1.12] | 0.90 [0.70, 1.10] | 0.693 |
| BUN (mg /dL) | 18.00 [13.00, 24.25] | 17.00 [14.00, 21.00] | 0.233 |
| Sodium (mmol/L) | 138.00 [136.00, 141.00] | 139.00 [137.00, 141.00] | 0.029 |
| Potassium (mmol/L) | 4.35 [4.10, 4.80] | 4.30 [3.90, 4.60] | 0.039 |
| Bicarbonate (mmol/L) | 22.00 [21.00, 24.00] | 23.00 [21.00, 24.00] | 0.005 |
| Time interval (h) | 3.56 [2.48, 5.08] | 3.49 [2.49, 4.85] | 0.553 |
| Outcomes |  |  |  |
| LOS in ICU (day) | 2.31 [1.33, 4.25] | 1.90 [1.25, 3.17] | <0.001 |
| LOS in hospital (day) | 10.54 [7.89, 14.85] | 7.91 [5.65, 10.93] | <0.001 |
| Hospital mortality (%) | 1 (1.0) | 12 (1.3) | 1 |
| Mortality 30‐day (%) | 2 (2.0) | 16 (1.8) | 0.359 |
| Mortality 90‐day (%) | 10 (10.0) | 28 (3.1) | 0.002 |

Values are expressed as median (interquartile range) or mean (standard deviation) and frequencies (percentages). AKI, acute kidney injury; BMI, body mass index; GCS, Glasgow coma scale; SOFA, sequential organ failure assessment; SPO_2_, oxyhemoglobin saturation by pulse oximetry; IABP, intra-aortic balloon pump; GNRI, geriatric nutritional risk index; PNI, prognostic nutritional index; BUN, blood urea nitrogen; LOS, length of stay.

**Table S3.** Characteristics of the included patients (stratified by PNI).

|  | Malnutrition (＜48) | Normal (≥ 48) | *p* value |
| --- | --- | --- | --- |
| n | 480 | 527 |  |
| Moderate to severe AKI | 286 (59.6) | 238 (45.1) | <0.001 |
| Gender (Male) | 344 (71.7) | 392 (74.4) | 0.368 |
| Age (year) | 75.21 [69.77, 80.73] | 72.49 [68.59, 76.83] | <0.001 |
| BMI (kg/m2) | 28.52 [25.51, 31.79] | 29.52 [26.22, 33.25] | 0.002 |
| Height (cm) | 170.00 [163.00, 178.00] | 170.00 [165.00, 178.00] | 0.245 |
| Weight (kg) | 82.85 [72.95, 93.50] | 86.00 [75.78, 98.88] | 0.001 |
| Comorbid disease |  |  |  |
| Charlson comorbidity index | 6.00 [4.00, 7.00] | 4.00 [3.00, 6.00] | <0.001 |
| Congestive heart failure | 185 (38.5) | 102 (19.4) | <0.001 |
| Peripheral vascular disease | 77 (16.0) | 76 (14.4) | 0.53 |
| Hypertension | 243 (50.6) | 345 (65.5) | <0.001 |
| Chronic pulmonary disease | 111 (23.1) | 101 (19.2) | 0.144 |
| Diabetes | 200 (41.7) | 227 (43.1) | 0.698 |
| Chronic liver disease | 8 (1.7) | 8 (1.5) | 1 |
| Stroke | 38 (7.9) | 37 (7.0) | 0.674 |
| Chronic kidney disease | 139 (29.0) | 88 (16.7) | <0.001 |
| Cerebrovascular disease | 59 (12.3) | 72 (13.7) | 0.581 |
| GCS | 14.00 [12.00, 15.00] | 14.00 [14.00, 15.00] | 0.001 |
| SOFA | 6.00 [4.00, 9.00] | 5.00 [4.00, 7.00] | <0.001 |
| Heart rate (beat) | 82.00 [77.00, 87.00] | 82.00 [76.00, 87.00] | 0.567 |
| MBP (mmHg) | 72.00 [68.00, 76.00] | 73.00 [70.00, 76.00] | <0.001 |
| SPO_2_ (%) | 98.00 [97.00, 99.00] | 98.00 [97.00, 99.00] | 0.008 |
| IABP | 52 (10.8) | 11 (2.1) | <0.001 |
| Ventilator | 420 (87.5) | 452 (85.8) | 0.476 |
| Vasopressor | 425 (88.5) | 451 (85.6) | 0.193 |
| Preoperative laboratories |  |  |  |
| Albumin (mg/dL) | 3.70 [3.40, 3.90] | 4.30 [4.00, 4.50] | <0.001 |
| Creatinine (mg/dL) | 1.10 [0.90, 1.40] | 1.00 [0.90, 1.20] | 0.002 |
| Hematocrit (%) | 35.10 [30.85, 39.18] | 39.30 [35.80, 42.00] | <0.001 |
| BUN (mg/dL) | 21.00 [17.00, 28.00] | 19.00 [16.00, 24.00] | <0.001 |
| Sodium (mmol/L) | 139.00 [137.00, 141.00] | 139.00 [138.00, 141.00] | 0.033 |
| Potassium (mmol/L) | 4.20 [3.90, 4.40] | 4.30 [4.00, 4.50] | 0.008 |
| Bicarbonate (mmol/L) | 26.00 [24.00, 28.00] | 26.00 [24.00, 28.00] | 0.002 |
| Lymphocyte(103/μL) | 1.32 [1.03, 1.68] | 1.94 [1.54, 2.46] | <0.001 |
| GNRI | 108.17 (11.56) | 119.83 (10.74) | <0.001 |
| PNI | 43.90 [40.98, 46.00] | 52.32 [49.98, 55.24] | <0.001 |
| Postoperative laboratories |  |  |  |
| Creatinine (mg/dL) | 0.90 [0.70, 1.20] | 0.90 [0.70, 1.00] | <0.001 |
| BUN (mg /dL) | 17.00 [14.00, 23.00] | 16.00 [13.00, 20.00] | 0.001 |
| Sodium (mmol/L) | 139.00 [137.00, 141.00] | 139.00 [137.00, 141.00] | 0.032 |
| Potassium (mmol/L) | 4.30 [3.90, 4.70] | 4.30 [4.00, 4.60] | 0.224 |
| Bicarbonate (mmol/L) | 22.50 [21.00, 24.00] | 23.00 [22.00, 24.00] | 0.013 |
| Time interval (h) | 3.65 [2.52, 4.85] | 3.39 [2.43, 4.89] | 0.286 |
| Outcomes |  |  |  |
| LOS in ICU (day) | 2.26 [1.30, 4.03] | 1.45 [1.21, 2.37] | <0.001 |
| LOS in hospital (day) | 9.86 [6.98, 12.95] | 6.74 [5.12, 9.83] | <0.001 |
| Hospital mortality (%) | 11 (2.3) | 2 (0.4) | 0.016 |
| Mortality 30‐day (%) | 12 (2.5) | 4 (0.8) | 0.051 |
| Mortality 90‐day (%) | 30 (6.2) | 8 (1.5) | <0.001 |

Values are expressed as median (interquartile range) or mean (standard deviation) and frequencies (percentages). AKI, acute kidney injury; BMI, body mass index; GCS, Glasgow coma scale; SOFA, sequential organ failure assessment; SPO_2_, oxyhemoglobin saturation by pulse oximetry; IABP, intra-aortic balloon pump; GNRI, geriatric nutritional risk index; PNI, prognostic nutritional index; BUN, blood urea nitrogen; LOS, length of stay.

**Table S4.** Multivariate logistic regression analysis of moderate to severe AKI in older patients undergoing CABG.

|  | B | SE | OR | CI | Z | *P* |
| --- | --- | --- | --- | --- | --- | --- |
| Gender | -0.456 | 0.165 | 0.63 | 0.46-0.88 | -2.76 | 0.006 |
| Congestive heart failure | 0.419 | 0.165 | 1.52 | 1.1-2.1 | 2.541 | 0.011 |
| Chronic pulmonary disease | 0.277 | 0.166 | 1.32 | 0.95-1.83 | 1.668 | 0.095 |
| SOFA | 0.069 | 0.027 | 1.07 | 1.02-1.13 | 2.583 | 0.01 |
| MBP | -0.056 | 0.013 | 0.95 | 0.92-0.97 | -4.224 | <0.001 |
| preoperative hematocrit | -0.023 | 0.012 | 0.98 | 0.95-1 | -1.815 | 0.07 |
| preoperative BUN | -0.051 | 0.017 | 0.95 | 0.92-0.98 | -3.032 | 0.002 |
| postoperative creatinine | 0.426 | 0.2 | 1.53 | 1.03-2.27 | 2.135 | 0.033 |
| postoperative BUN | 0.048 | 0.021 | 1.05 | 1.01-1.09 | 2.311 | 0.021 |

CABG, coronary artery bypass grafting; B, regression coefficient; SE, Standard error; OR, odds ratio; CI, confidence interval; SOFA, Sequential organ failure assessment; MBP, mean blood pressure; BUN, blood urea nitrogen.

**Table S5.** Comparative analysis of the discrimination of each nutritional index for postoperative modern to severe AKI in older patients undergoing CABG.

|  | AUC | | NRI | | IDI | |
| --- | --- | --- | --- | --- | --- | --- |
|  | Difference in AUC (95%) | *P* | Index (95%) | *P* | Index (95%) | *P* |
| Nutritional indices as continuous variables | | | | | | |
| model1 as the base model |  |  |  |  |  |  |
| +GNRI vs +PNI | 0.005 | 0.798 | -0.0177 (-0.074 - 0.038) | 0.535 | 0.008 (-0.007 - 0.023) | 0.298 |
| model2 as the base model |  |  |  |  |  |  |
| +GNRI vs +PNI | -0.011 | 0.393 | -0.0728 (-0.135 - -0.011) | 0.021 | -0.010 (-0.024 - 0.005) | 0.199 |
| model3 as the base model |  |  |  |  |  |  |
| +GNRI vs +PNI | -0.006 | 0.663 | -0.0092 (-0.067 - 0.048) | 0.753 | -0.004 (-0.018 - 0.009) | 0.539 |
| Nutritional indices as category variables | | | | | | |
| model1 as the base model |  |  |  |  |  |  |
| +GNRI vs +PNI | 0.011 | 0.319 | -0.006 (-0.042 - 0.031) | 0.753 | 0.010 (0.003 - 0.018) | 0.007 |
| model2 as the base model |  |  |  |  |  |  |
| +GNRI vs +PNI | 0.001 | 0.864 | 0.003 (-0.038 - 0.043) | 0.902 | 0.002 (-0.004 - 0.007) | 0.537 |
| model3 as the base model |  |  |  |  |  |  |
| +GNRI vs +PNI | 0.002 | 0.701 | -0.022 (-0.060 - 0.016) | 0.254 | 0.003 (-0.003 - 0.007) | 0.344 |

Multivariable analysis model 1: adjusting for gender, congestive heart failure and chronic pulmonary disease. Multivariable analysis model 2: adjusting for variables in model 1 as well as SOFA score, MBP, preoperative hematocrit and BUN. Multivariable analysis model 3: adjusting for variables in model 2 as well as postoperative creatinine and BUN. GNRI, geriatric nutritional risk index; PNI, prognostic nutritional index; AKI, acute kidney injury; CABG, coronary artery bypass grafting; AUC, area under curve; NRI, Net reclassification improvement; IDI, Integrated discrimination improvement.


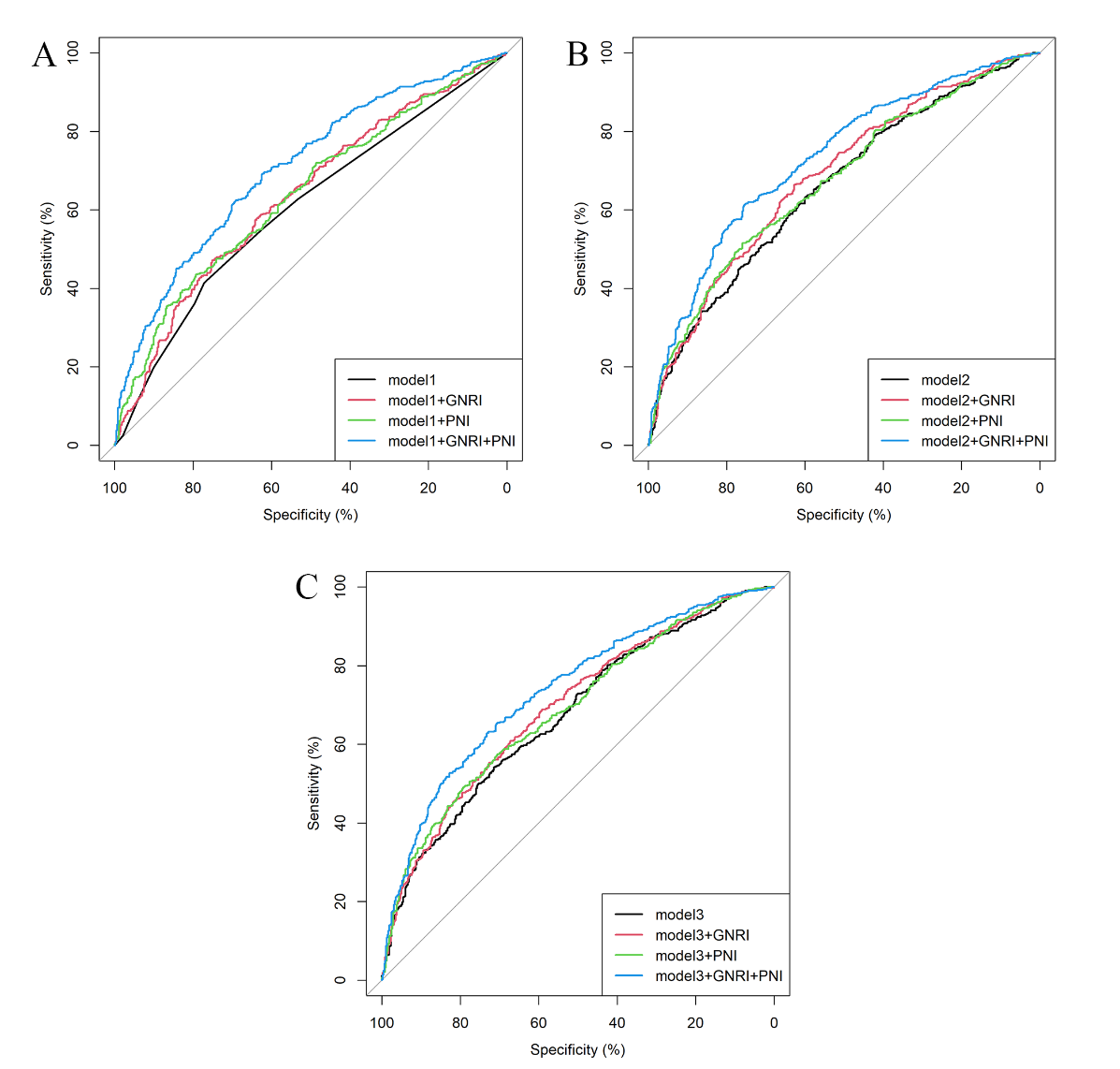


**Figure S1.** ROC of different models. The AUC of utilizing models with or without nutritional scores (as continuous variables) predicts the incidence of moderate to severe AKI in older patients undergoing CABG surgery. (A) Model 1 adjusted for demographic factors including gender, congestive heart failure, and chronic pulmonary disease; (B) Model 2 expanded on Model 1 by incorporating preoperative conditions such as the SOFA score, MBP, preoperative hematocrit, and BUN; (C) Model 3 further included postoperative creatinine and BUN levels. ROC, receiver operating characteristic curves; AUC, area under curve; AKI, acute kidney injury; CABG, coronary artery bypass graft; SOFA, sequential organ failure assessment; MBP, mean blood pressure; BUN, blood urea nitrogen; GNRI, geriatric nutritional risk index; PNI, prognostic nutritional index.


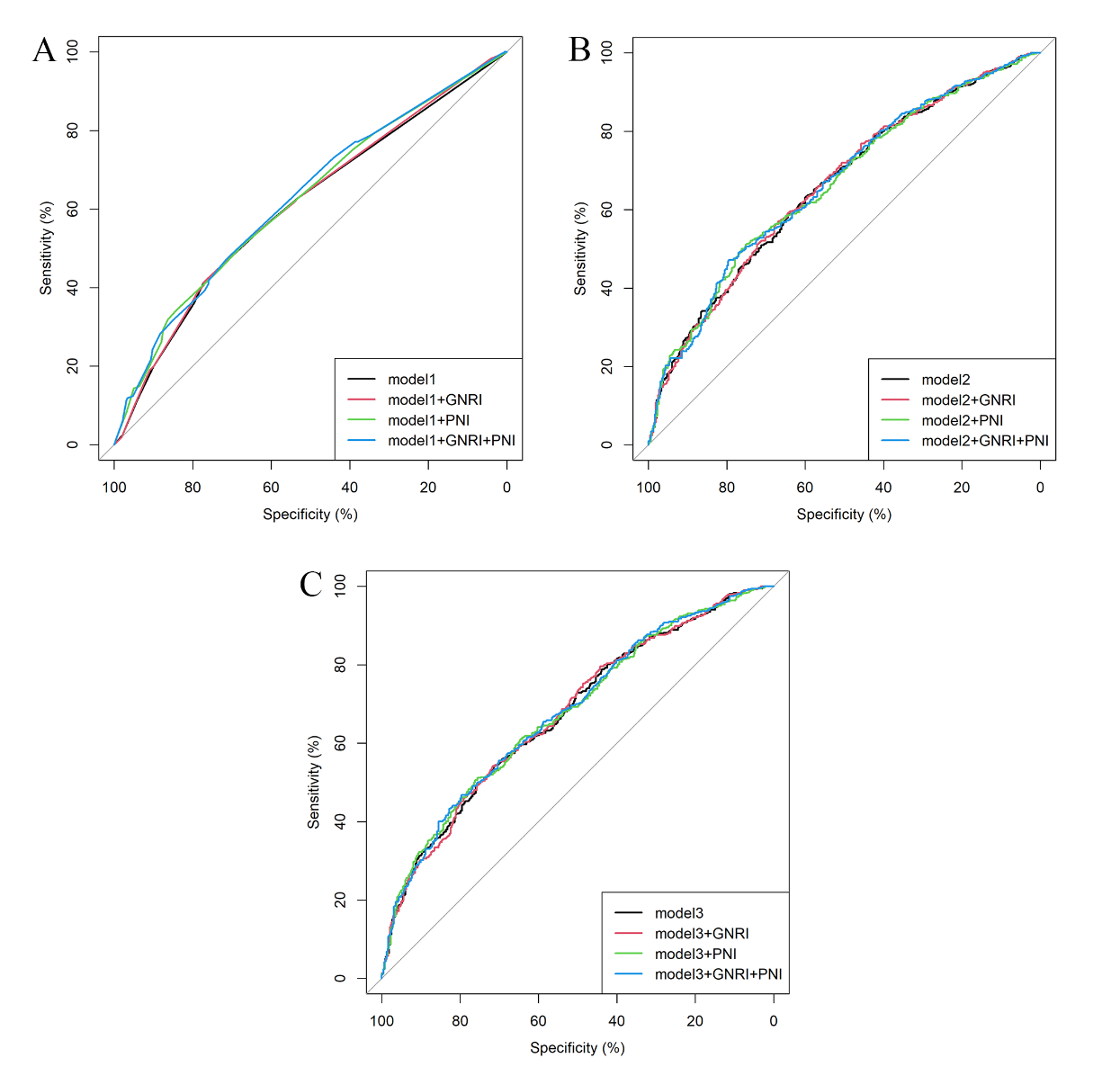


**Figure S2.** ROC of different models. The AUC of utilizing models with or without nutritional scores (as categorical variables) predicts the incidence of moderate to severe AKI in older patients undergoing CABG surgery. (A) Model 1 adjusted for demographic factors including gender, congestive heart failure, and chronic pulmonary disease; (B) Model 2 expanded on Model 1 by incorporating preoperative conditions such as the SOFA score, MBP, preoperative hematocrit, and BUN; (C) Model 3 further included postoperative creatinine and BUN levels. ROC, receiver operating characteristic curves; AUC, area under curve; AKI, acute kidney injury; CABG, coronary artery bypass graft; SOFA, sequential organ failure assessment; MBP, mean blood pressure; BUN, blood urea nitrogen; GNRI, geriatric nutritional risk index; PNI, prognostic nutritional index.


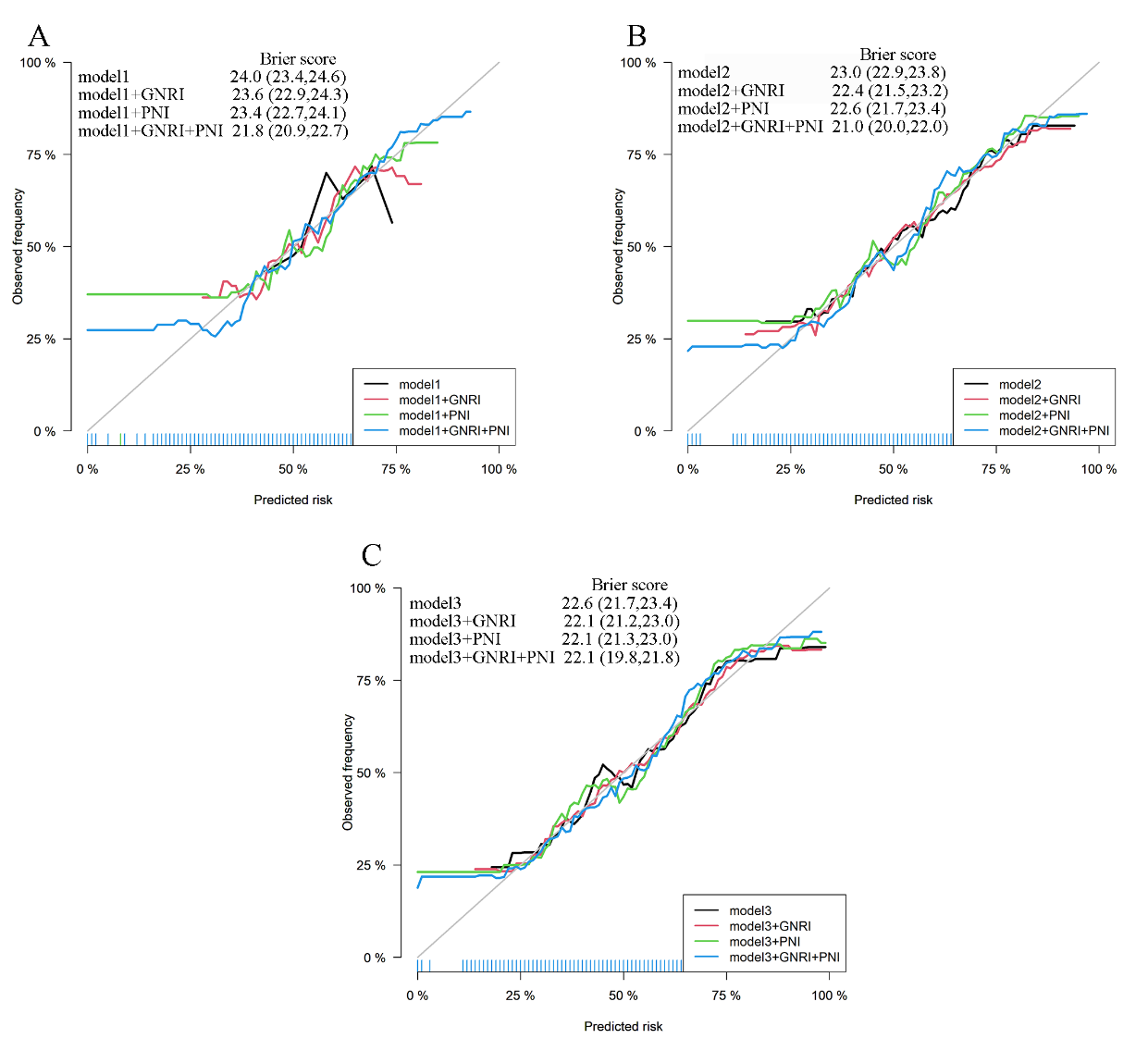


**Figure S3.** Calibration curves of the model calibration performance of model 1(A), model 2(B), model 3(C). Nutritional scores were regarding as continuous variables. Model 1 adjusted for demographic factors including gender, congestive heart failure, and chronic pulmonary disease; Model 2 expanded on Model 1 by incorporating preoperative conditions such as the SOFA score, MBP, preoperative hematocrit, and BUN; Model 3 further included postoperative creatinine and BUN levels. Brier score indicates goodness of fit. As Brier scores get smaller, goodness of fit increases. SOFA, sequential organ failure assessment; MBP, mean blood pressure; BUN, blood urea nitrogen; GNRI, geriatric nutritional risk index; PNI, prognostic nutritional index.


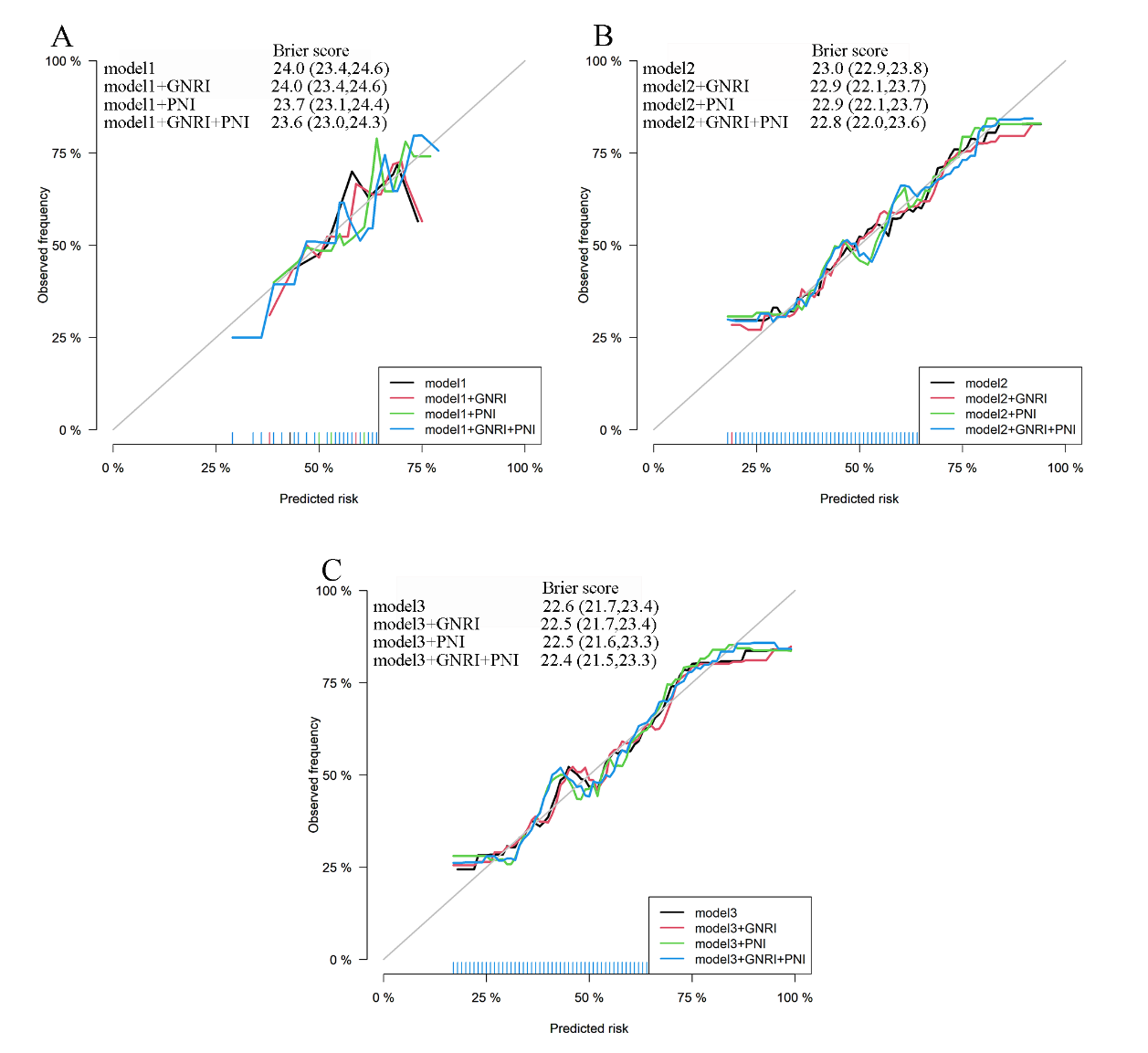


**Figure S4.** Calibration curves of the model calibration performance of model 1(A), model 2(B), model 3(C). Nutritional scores were regarding as categorical variables. Model 1 adjusted for demographic factors including gender, congestive heart failure, and chronic pulmonary disease; Model 2 expanded on Model 1 by incorporating preoperative conditions such as the SOFA score, MBP, preoperative hematocrit, and BUN; Model 3 further included postoperative creatinine and BUN levels. Brier score indicates goodness of fit. As Brier scores get smaller, goodness of fit increases. SOFA, sequential organ failure assessment; MBP, mean blood pressure; BUN, blood urea nitrogen; GNRI, geriatric nutritional risk index; PNI, prognostic nutritional index.


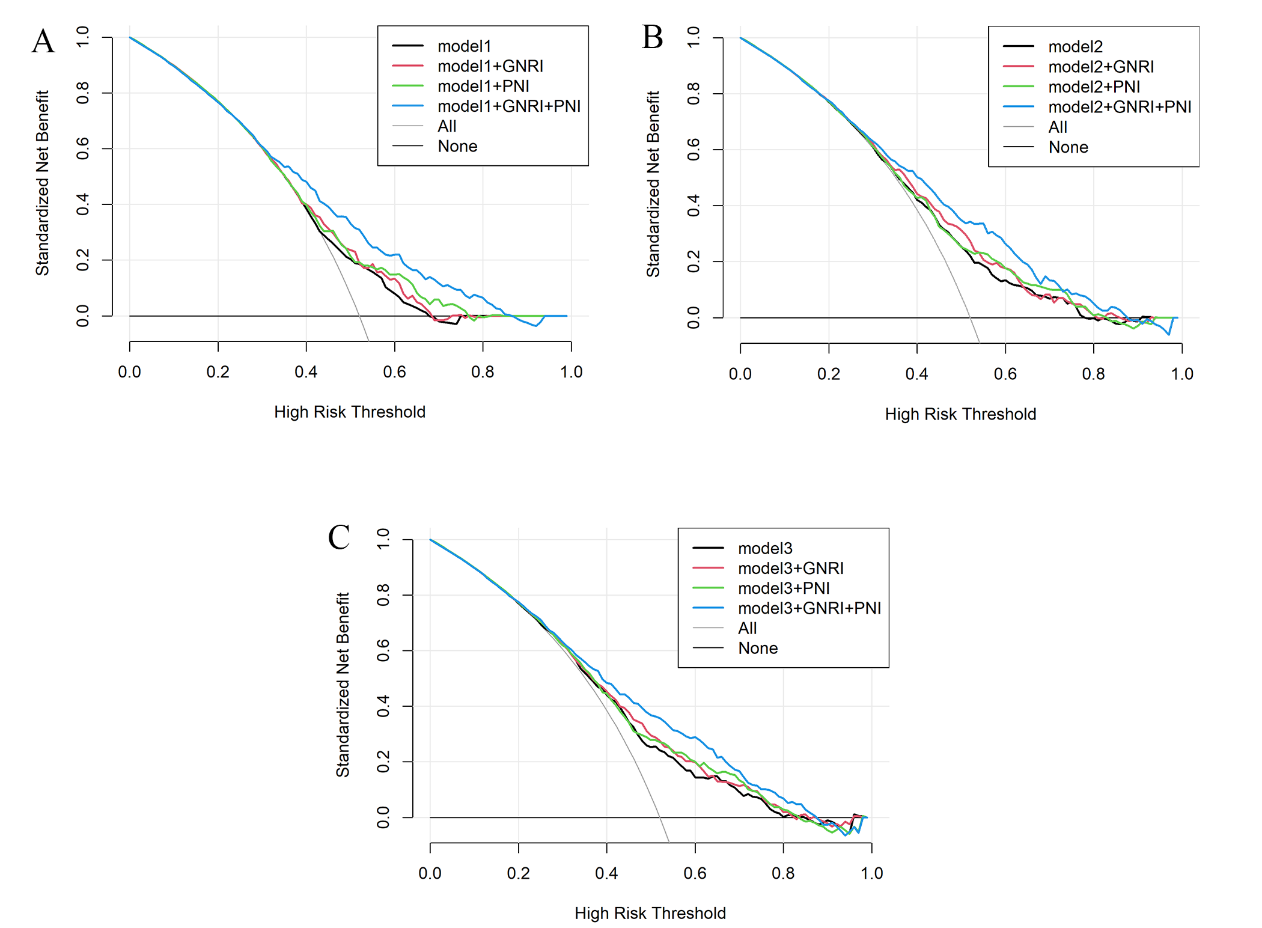


**Figure S5.** Decision curve analysis. The net benefit of utilizing models with or without nutritional scores (as continuous variables) for prophylactic treatment decisions for postoperative moderate to severe AKI. (A) Base model 1 adjusted for demographic factors including gender, congestive heart failure, and chronic pulmonary disease; (B) Base model 2 expanded on Model 1 by incorporating preoperative conditions such as the SOFA score, MBP, preoperative hematocrit, and BUN; (C) Base model 3 further included postoperative creatinine and BUN levels. SOFA, sequential organ failure assessment; MBP, mean blood pressure; BUN, blood urea nitrogen; GNRI, geriatric nutritional risk index; PNI, prognostic nutritional index.


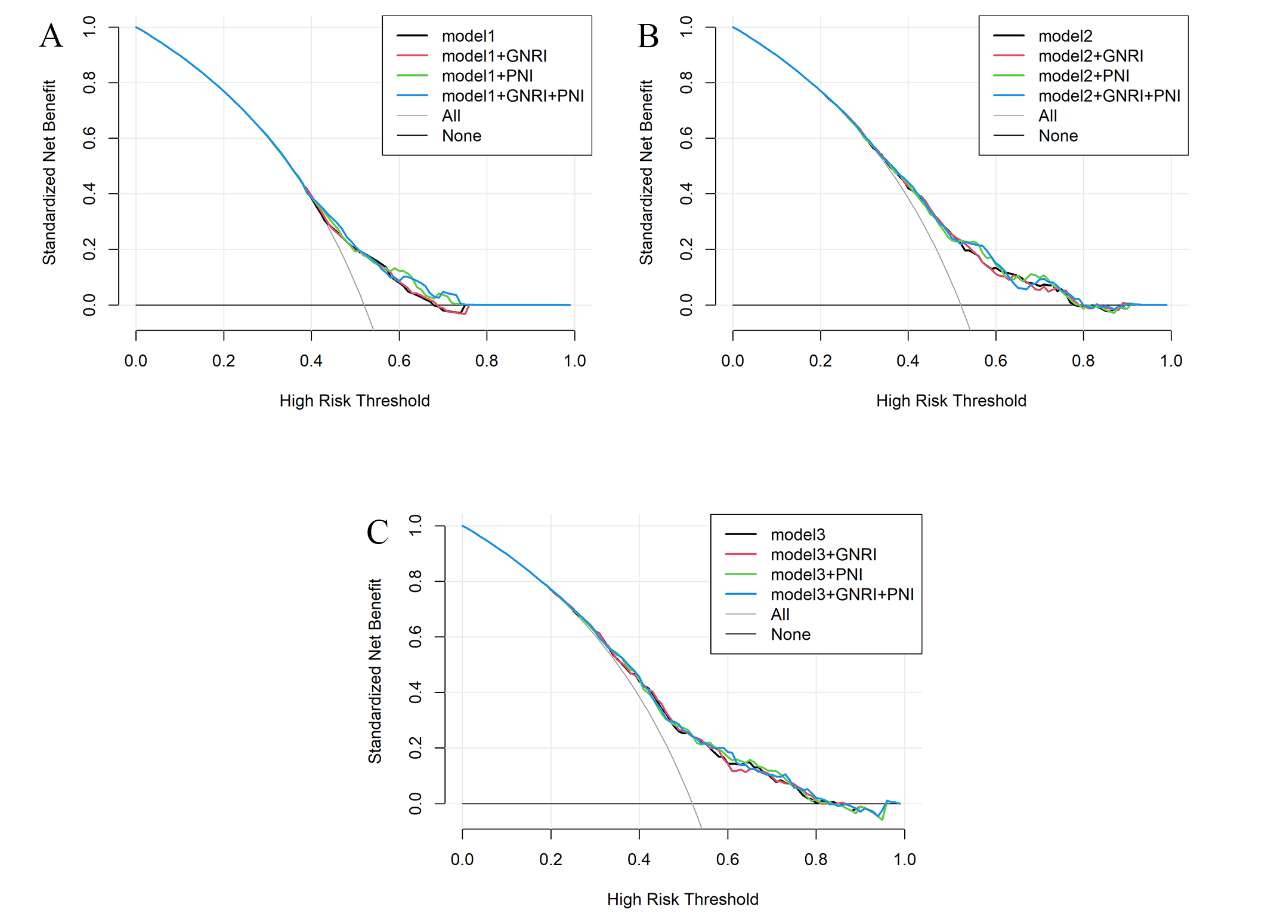


**Figure S6.** Decision curve analysis. The net benefit of utilizing models with or without nutritional scores (as categorical variables) for prophylactic treatment decisions for postoperative moderate to severe AKI. (A) Base model 1 adjusted for demographic factors including gender, congestive heart failure, and chronic pulmonary disease; (B) Base model 2 expanded on Model 1 by incorporating preoperative conditions such as the SOFA score, MBP, preoperative hematocrit, and BUN; (C) Base model 3 further included postoperative creatinine and BUN levels. SOFA, sequential organ failure assessment; MBP, mean blood pressure; BUN, blood urea nitrogen; GNRI, geriatric nutritional risk index; PNI, prognostic nutritional index.
